# Metazoans of redoxcline sediments in Mediterranean deep-sea hypersaline anoxic basins

**DOI:** 10.1186/s12915-015-0213-6

**Published:** 2015-12-10

**Authors:** Joan M. Bernhard, Colin R. Morrison, Ellen Pape, David J. Beaudoin, M. Antonio Todaro, Maria G. Pachiadaki, Konstantinos Ar. Kormas, Virginia P. Edgcomb

**Affiliations:** Geology & Geophysics Department, Woods Hole Oceanographic Institution, Woods Hole, MA USA; Biology Department, University of Nevada Reno, Reno, NV USA; Marine Biology Research Group, Ghent University, Ghent, Belgium; Biology Department, Woods Hole Oceanographic Institution, Woods Hole, MA USA; Department of Life Sciences, University of Modena & Reggio Emilia, Modena, Italy; Department of Ichthyology & Aquatic Environment, School of Agricultural Sciences, University of Thessaly, Volos, Greece

**Keywords:** Athalassohaline, Bryozoa, CellTracker^TM^ Green, Discovery, L’Atalante, Loricifera, Meiofauna, Nematoda, Ultrastructure, Urania

## Abstract

**Background:**

The deep-sea hypersaline anoxic basins (DHABs) of the Mediterranean (water depth ~3500 m) are some of the most extreme oceanic habitats known. Brines of DHABs are nearly saturated with salt, leading many to suspect they are uninhabitable for eukaryotes. While diverse bacterial and protistan communities are reported from some DHAB haloclines and brines, loriciferans are the only metazoan reported to inhabit the anoxic DHAB brines. Our goal was to further investigate metazoan communities in DHAB haloclines and brines.

**Results:**

We report observations from sediments of three DHAB (Urania, Discovery, L’Atalante) haloclines, comparing these to observations from sediments underlying normoxic waters of typical Mediterranean salinity. Due to technical difficulties, sampling of the brines was not possible. Morphotype analysis indicates nematodes are the most abundant taxon; crustaceans, loriciferans and bryozoans were also noted. Among nematodes, *Daptonema* was the most abundant genus; three morphotypes were noted with a degree of endemicity. The majority of rRNA sequences were from planktonic taxa, suggesting that at least some individual metazoans were preserved and inactive. Nematode abundance data, in some cases determined from direct counts of sediments incubated in situ with CellTracker^TM^ Green, was patchy but generally indicates the highest abundances in either normoxic control samples or in upper halocline samples; nematodes were absent or very rare in lower halocline samples. Ultrastructural analysis indicates the nematodes in L’Atalante normoxic control sediments were fit, while specimens from L’Atalante upper halocline were healthy or had only recently died and those from the lower halocline had no identifiable organelles. Loriciferans, which were only rarely encountered, were found in both normoxic control samples as well as in Discovery and L’Atalante haloclines. It is not clear how a metazoan taxon could remain viable under this wide range of conditions.

**Conclusions:**

We document a community of living nematodes in normoxic, normal saline deep-sea Mediterranean sediments and in the upper halocline portions of the DHABs. Occurrences of nematodes in mid-halocline and lower halocline samples did not provide compelling evidence of a living community in those zones. The possibility of a viable metazoan community in brines of DHABs is not supported by our data at this time.

**Electronic supplementary material:**

The online version of this article (doi:10.1186/s12915-015-0213-6) contains supplementary material, which is available to authorized users.

## Background

The remarkable recent description of certain metazoans that complete a full life cycle without access to dissolved oxygen [1] has attracted considerable scientific and public interest. Three new species of loriciferans collected in sediments from a deep hypersaline anoxic basin (DHAB) were reported to have intact living tissue implying viability. Viability of these loriciferans was asserted based on a series of analyses [1]. Although it has been known for many decades that metazoans inhabit anoxic (complete lack of dissolved oxygen) habitats either on a periodic, transient, or semi-permanent basis (reviewed in [2]), none had been shown to complete an entire life cycle in such an environment. The dogma was that, until that report, animals required oxygen in at least part of their life cycle. Thus, Danovaro et al. [1] raised many intriguing questions pertaining to metazoan physiology and evolution [3].

Aside from the Danovaro et al. [1] publication, reports of anaerobic metazoans have long existed. Periodic exposures to anoxia can even occur in muscle tissue during exercise, for example, or during low tide exposure when organisms such as mussels close valves to prevent dehydration. Longer exposures to anoxia can occur on seasonal scales when lakes ice over, for instance. A number of metazoan intestinal-tract parasites (e.g. cestodes, certain nematodes) can live in the nearly anoxic intestinal habitat for significant periods of their life cycle. Digestive tracts of metazoa rarely become truly anoxic, given diffusion of oxygen through the gut lining. We focus the remainder of our discussion on free-living metazoans.

Meiofaunal metazoans (in general, those that pass through a 1-mm screen but are retained on a ~50-μm screen), such as certain nematodes and oligochaetes, reportedly live in sediments where oxygen is undetectable (e.g. [2, 4–6]). While it is possible that these metazoa live without oxygen, typically in nature they can also relatively easily access oxygen by migrating in the sediment toward oxic habitats. Regardless, certain taxa of metazoan meiofauna appear to survive extended periods without oxygen and/or can complete their entire life cycle with only trace concentrations of oxygen (e.g. see discussion in [2]).

In addition to a complete lack of oxygen or only the presence of trace oxygen, subsurface sediments typically have high concentrations of hydrogen sulfide. Sulfide binds to cytochrome c oxidase, which is a crucial enzyme in aerobic respiration, inhibiting its normal function. In brief, this observation spawned the dogma that aerobes cannot survive long periods of exposure to sulfidic conditions. Of course, there are biochemical adaptations that permit exceptions, including the ability of some mitochondria to oxidize sulfide (e.g. [7–9]). Certain metazoan species seem to aggregate in the sulfidic zone, implying that sulfide is not harmful to their survival (e.g. [10]). Importantly, to our knowledge, no metazoan that lacks electron transport phosphorylation, which is the pervasive, ATP-producing pathway in mitochondria, has been identified, although a number of metazoans use compounds other than oxygen as their terminal electron acceptor, fumarate being perhaps the best studied example [11]. For further discussion of eukaryotic anaerobic metabolic pathways, see Müller et al. [11] and references therein, as the details of metazoan biochemistry in anoxic environments are not a focus of this contribution.

Herein, we report results of visually-guided sediment coring on the metazoan assemblages in DHAB halocline and adjacent normoxic, normal saline (control) sediments, providing additional information on whether observed metazoa represent living eukaryotic populations. Our attempts to sample beneath full strength brines were unsuccessful.

### The DHAB environment

**Fig. 1:**
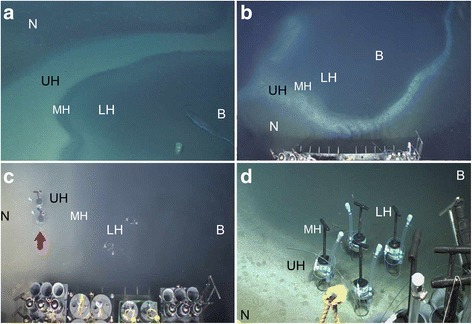
Underwater photographs of representative deep hypersaline anoxic basin halocline interfaces. **a** Discovery showing floating garbage (aluminum beverage can and plastic item) atop brine. **b** Portion of L’Atalante halocline showing embayment. **c** Urania showing emplaced injector cores (607-608cC and 607-608cD on left; E and F on right). Note the impaired visibility of cores on the right due to the brine (B) murkiness. Cores C and D are in the normoxic control/halocline transition (red arrow). **d** L’Atalante halocline with emplaced injector cores (611cC and 611cF in the upper halocline (UH) zone; 611cA and 611cB in the mid-halocline (MH)). LH, Lower halocline; N, Normoxic normal saline. Outer diameter of pushcorer is 6.9 cm

Eastern Mediterranean DHABs, which were first described about three decades ago [12], are one of the most extreme marine environments presently known to science. These basins are characterized by nearly saturated salt concentrations, depending on the basin, as well as variable concentrations of sulfide and other ions [13]. Located at a depth of more than 3000 m below sea level, the brines enclosed in these geographically isolated basins are characterized by high pressure and anoxic conditions. The water activity (a measure of the energy status of the water in a system) is extremely low. For example, in the brine of Discovery Basin (35°17.15N, 21°42.31E) it is so low that this and similar DHABs are some of the driest places on Earth despite being located in the ocean [14]. There are varied dates postulated for the origin of these DHABs [15, 16]. L’Atalante is determined to be the oldest of the three DHABs we studied; at most, it is 53,000 years old [15]. The Eastern Mediterranean DHABs formed from the dissolution of subterranean Miocene salt deposits and/or the release of trapped brines that were exposed to seawater after tectonic activity [17]. The high density of DHAB brines presumably prevent mixing with overlying oxygenated seawater, resulting in a sharp halocline of about 2-m thickness (Fig. 1). Because we sampled with a Remotely Operated Vehicle (ROV) (see Methods), we could target visible zones where the halocline impinged the seafloor (Fig. 1).

Each of the three DHABs we studied (Urania, Discovery, L’Atalante) is geochemically distinct (Table 1), although some similarities exist between L’Atalante and Urania brine chemistries [13]. Discovery is athalassohaline, with a vastly different ionic composition from seawater; L’Atalante and Urania are thalassohaline, with ionic compositions similar to seawater. The Na^+^ concentrations in L’Atalante and Urania brines (4235 mM and 3503 mM, respectively) are much higher than in the Discovery brine (68 mM) and seawater (528 mM). The highest concentration of MgCl_2_ found in a marine environment to date occurs in the Discovery brine (4995 mM Mg^+^ vs. 410 mM in L’Atalante and 316 mM in Urania) [13]. The concentration of sulfide in Urania brine is very high (16 mM), approximately 16 times higher than in Discovery, >5 times that of L’Atalante brine, and orders of magnitude more concentrated than that of typical seawater (2.6 × 10^−6^ mM). Urania is unique among the DHABs studied here in that it has a hydrothermal mud vent in the western sector [18], relatively near our sampling location. Mud fluids are less saline than the overlying brine [18], but brine and mud fluids do not mix due to the higher density of the mud-laden fluids. The degree to which mud fluids extend to our sampling site is not known, but porewaters could have a salinity of ~100 rather than the brine salinity of ~270 [18].

**Table 1:** Geochemical data of deep hypersaline anoxic basin (DHAB) brines and typical seawater (modified from [13, 21])

Geochemistry	L’Atalante	Discovery	Urania	Seawater
Coordinates (N)	35°18.87	35°17.15	35°13.78	–
(E)	21°24.34	21°42.31	21°28.94	–
Water depth (m)	3430	3582	3468	–
Density (10^3^ kg m^−3^)	1.23	1.35	1.13	1.03
Na^+^ (mM)	4674	68	3503	528
Cl^−^ (mM)	5289	9491	3729	616
Mg^2+^ (mM)	410	4995	316	60
K^+^ (mM)	369	20	122	11
SO_4_^2−^ (mM)	397	96	107	32
HS^−^ (mM)	2.9	1	16	2.6 × 10^−6^
CH_4_ (mM)	0.5	0.03	5.6	1.4 × 10^−6^

Coordinates (degrees (°) decimal minutes) and water depths reflect our approximate sampling area for each DHAB

Dissolved oxygen can remain present in upper halocline waters (e.g. [19, 20]). Oxygen can be detectable in pore waters to depths of 1–2 cm in upper halocline sediments and to depths of ~2–5 mm in lower halocline sediments [21]. In one lower halocline pushcore (Urania, 608c11), oxygen was near the detection limit throughout the top 2.5 cm as well as in the overlying waters. In sum, dissolved oxygen in DHAB upper to middle halocline sediments is generally detectable in the surface millimeters yet has steep gradients.

Diverse communities of bacteria and protists have been documented in the water columns of these basins (e.g. [13, 14, 19, 20, 22–25]). Some bacteria, archaea, protists, and fungi have also been detected in DHAB sediments (e.g. [21, 26, 27]).

## Results

### Metazoan community: morphotypes

While nematodes were the most common metazoan group present in our samples from a morphological perspective, additional metazoan morphotypes were also occasionally observed in our materials. We focus detailed discussion on the Nematoda in most subsequent sections (see below).

**Fig. 2:**
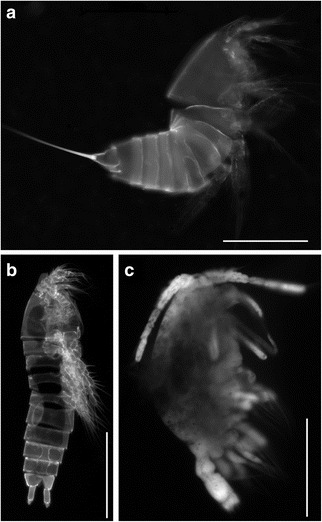
DAPI-labeled copepod crustaceans viewed with epifluorescence microscopy and DAPI optics (377-nm excitation; 447-nm emission). **a** Molted exoskeleton of a fifth-stage harpacticoid copepodite (L’Atalante lower halocline, 611c14). **b** Intact fifth-stage harpacticoid copepodite (Discovery lower halocline, 609c14). **c** Mid- to late-stage cyclopoid copepodite (Discovery lower halocline, 610c9). Note lack of strong DAPI signal in all but appendages of (**c**). Scales: **a**, **c** = 100 μm; **b** = 200 μm

Crustaceans, all copepods, were observed occasionally in our sediment samples (Fig. 2). In some cases, the exoskeleton molt of a harpacticoid copepodite (Fig. 2a) was observed. In other cases, copepod specimens appeared intact (Fig. 2b, c), but none appeared to have copious amounts of internal tissue. When 4’,6-diamidino-2-phenylindole (DAPI)-stained (a DNA intercalating fluorochrome) copepods were viewed with appropriate filter sets, those collected from the halocline did not appear to be well labeled (Fig. 2a, b), although some specimens (e.g. Fig. 2c) had appendages that appeared to have labeled with DAPI. Harpacticoida, which typically have a benthic life habit, were the most common copepod taxon encountered.

**Fig. 3:**
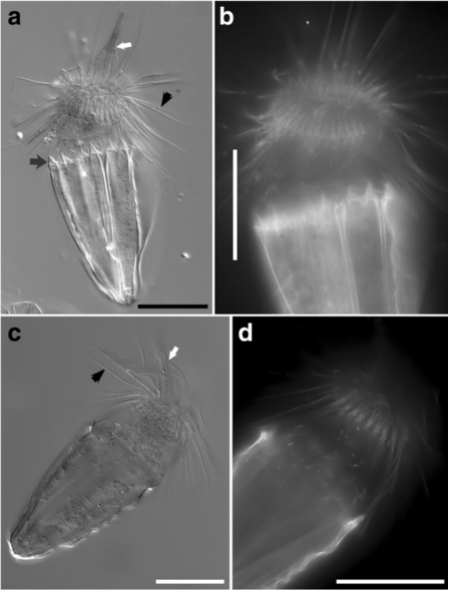
Loriciferan *Spinoloricus cinziae*, from L’Atalante imaged with differential interference contrast (DIC) (**a**, **c**) and DAPI (**b**, **d**). **a**, **b** Specimen from normoxic control sediments (611c3). Mouth cone (white arrow), lorica spines (black arrow), and scalids (black arrowhead) are visible in (**a**). Further images are shown in Additional file 1. **c**, **d** Specimen from L’Atalante lower halocline sediments (611c14). Mouth cone (white arrow) and scalids (black arrowhead) are visible in (**c**). Further images are shown in Additional file 2. Scales: **a**–**d** = 50 μm

**Fig. 4:**
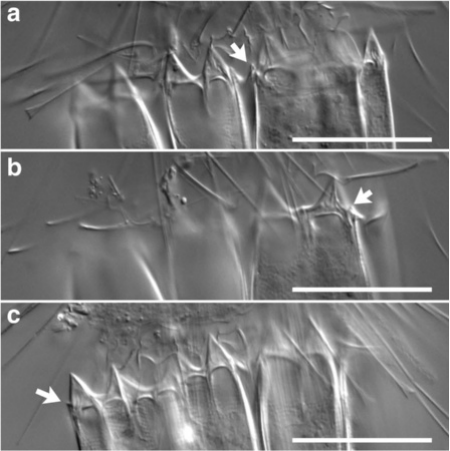
*Spinoloricus cinziae*, details of the lorica. **a**–**c** Photos at different focal planes refer to specimen shown in Fig. 3a, b. Arrows indicate the extra spines of the lorica. Scales: **a**–**c** = 30 μm

**Fig. 5:**
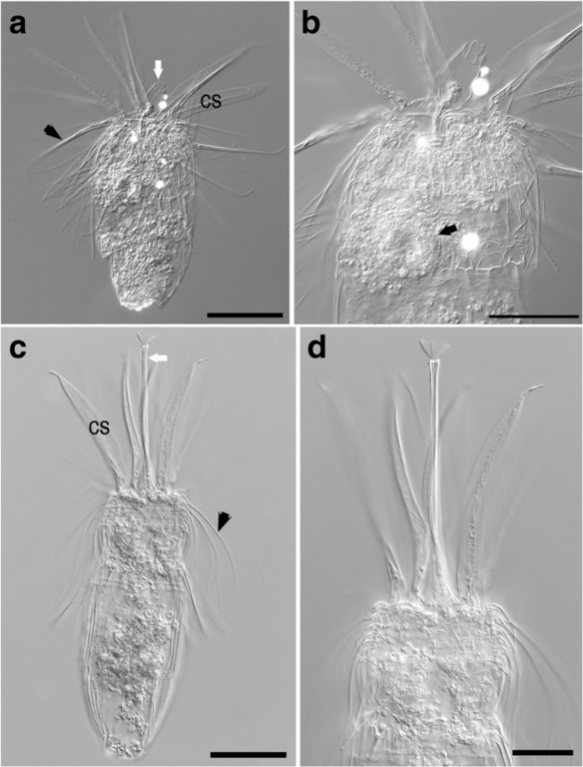
Loriciferans. **a**, **b** *Rugiloricus* sp., imaged with differential interference contrast (DIC), from L’Atalante lower halocline sediments (611c17). **a** Overview showing mouth cone (white arrow) and clavoscalid (cs). **b** Higher magnification view showing putative oocyte (black arrow). Further images are shown in Additional file 3. **c**, **d** *Pliciloricus* sp., imaged with DIC, from L’Atalante lower halocline sediments (611c17). **c** Overview showing scalids (black arrowhead), clavoscalids (cs), and mouth cone (white arrow). **d** Higher magnification of the anterior region (introvert). Further images are shown in Additional file 4. Scales: **a**, **c** = 50 μm; **b** = 30 μm; **d** = 25 μm

Loriciferan specimens were only rarely observed, compared to nematodes (Figs. 3, 4, 5 and Additional files 1, 2, 3, 4, 5, 6). A total of 16 specimens were observed during the entire project (~295 cm^3^ in situ seafloor volume). These individuals were obtained from both L’Atalante (n = 10) and Discovery (n = 5) lower halocline samples (samples 611c17, 611c14 and 609c14, respectively; Figs. 3c, d, 4, and 5a–d) as well as from a normoxic, normal saline control (n = 1) sample (611c3; Figs. 3a, b and 4). Three loriciferan morphotypes were observed in the L’Atalante lower halocline: *Spinoloricus cinziae* [28], *Rugiloricus* sp., and an additional species potentially belonging to the genus *Pliciloricus* (611c14; 611c17) (see more on this below). *Rugiloricus* sp. was also observed in a Discovery lower halocline sample (609c14) and *S. cinziae* was also observed in an L’Atalante control sample (611c3).

Only two loriciferan specimens, one each from L’Atalante normoxic control and lower halocline, were detected in the Percoll density-gradient material incubated with DAPI; neither specimen had convincing DAPI label in their metazoan tissues (Fig. 3b, d; compare to Fig. 9e–h). While neither of the DAPI-stained specimens exhibited convincing reproductive structures, each of two Rose Bengal stained loriciferans displayed what can be interpreted as an oocyte (L’Atalante lower halocline, 611c17, Fig. 5b; Discovery lower halocline, 609c14; Additional file 6). There were, however, no identifiable internal organs in any of the loriciferans examined at high magnification (n = 4).

**Fig. 6:**
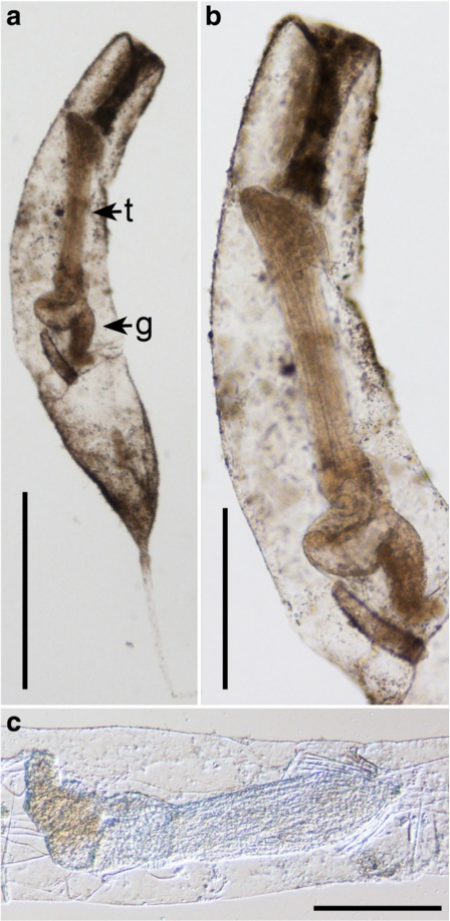
Bryozoan. *Ctenostomata* sp. from Urania normoxic, normal saline/halocline transition (607-608cC). **a**, **b** Bright field images showing same individual at different magnifications. **c** Differential interference contrast image showing internal anatomy of a different specimen. t, Tentacles; g, Gut. Scales: **a** = 400 μm; **b**, **c** = 200 μm

One sample from the transition between normoxic, normal saline sediments and the upper halocline of Urania (607-608cC; Fig. 1c) had numerous specimens of what appear to be a single bryozoan morphotype (Fig. 6). The morphotype is not calcified, perhaps belonging to the ctenostome genus *Triticella* [29].

### Metazoan community: molecular signatures

Materials from five cores were subject to sequencing: one normoxic, normal saline control sample was analyzed from Urania, as were Urania halocline, upper haloclines from L’Atalante and Discovery, and the lower halocline of L’Atalante. The vast majority of ribosomal RNA (rRNA) sequences from this dataset were of unicellular eukaryotes (protists and fungi), which are presented in Bernhard et al. [21]. The rRNA sequences of metazoan groups were dominated by crustaceans (Table 2). The dominant crustacean group was calanoid copepods (7 of 10 crustacean operational taxonomic units (OTUs) at 97 % sequence similarity). In addition, another relatively abundant group was affiliated with Maxillopoda. This group included OTUs with 91–98 % sequence similarity to known metazoa in GenBank, including five copepod sequences, two of which were calanoid sequences, and OTUs of undetermined affiliation. The L’Atalante upper halocline had the highest detected metazoan diversity, with sequences from all but two of the groups detected by sequencing (*Neocalanus*, *Pontophilus*). Signatures of the shrimp *Pontophilus* were detected solely in the Discovery upper halocline sample; no other metazoan sequences were recovered from this sample. The normoxic control sample had low OTU diversity with only three metazoan taxa represented: two calanoid copepods plus an additional, undetermined OTU affiliated with Maxillopoda. In addition to the copepod sequences, genetic signatures from three types of Cnidaria and one Ctenophora were recovered from the L’Atalante upper halocline sample. The sequences have been deposited in the GenBank SRA archive under the accession number SRP049010. A mapping file showing how to link sequence identifiers with environmental source (basin and habitat) is provided in Additional file 7.

**Table 2:** Relative abundance of metazoan small subunit ribosomal (SSU) RNA retrieved, calculated as percentage of total eukaryotic SSU RNA reads obtained per library

Higher taxon	Lower taxon	L UH	U H	U C	D UH	L LH
Crustacea	*Centropages*	0.72	0	1.27	0	0
Crustacea	*Clausocalanus*	0.24	0	0	0	0
Crustacea	*Labidocera*	0.72	0	0	0	0
Crustacea	*Neocalanus*	0	0	2.53	0	0
Crustacea	*Paraeucalanus*	0.24	0	0	0	0
Crustacea	*Pontella*	0.24	0	0	0	0.37
Crustacea	*Subeucalanus*	0.24	0	0	0	0.91
Crustacea	Maxillopoda	2.39	1.20	1.27	0	0.18
Crustacea	*Oithona*	0.24	0	0	0	0
Crustacea	*Pontophilus*	0	0	0	0.02	0
Cnidaria	*Aglaura*	0.48	0	0	0	0
Cnidaria	Hydrozoa	0.48	0	0	0	0
Cnidaria	*Pantachogon*	0.24	0	0	0	0
Ctenophora	Undescribed	0.24	0	0	0	0

Taxonomic assignments were made using BLAST against the PR2 database within QIIME. L, L’Atalante; U, Urania; D, Discovery; UH, Upper halocline; H, Halocline; LH, Lower halocline; C, Normoxic, normal saline control

Curiously, no nematode sequences were recovered in our eukaryotic small subunit rRNA dataset even though the vast majority of metazoan morphotypes obtained were nematodes, and transmission electron microscopy (TEM) imaging results indicated living nematodes in the control sediments at the very least (see below). No loriciferan sequences were recovered either.

### Nematode morphotype identifications

**Fig. 7:**
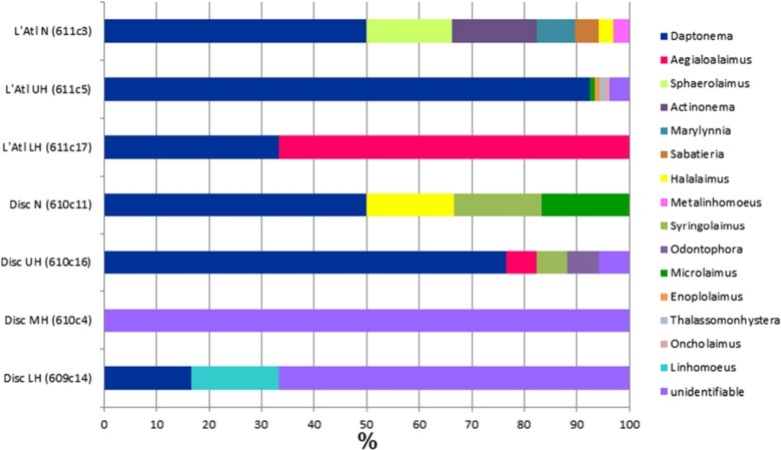
Relative abundances (%) of nematode genera in sediment samples, as determined by morphotype analyses. L’Atalante (L’Atl); Discovery (Disc). Parenthetic values represent dive and core number. N, Normoxic, normal saline control; UH, Upper halocline; LH, Lower halocline; MH, Mid-halocline

Fifteen nematode genera were identified in a suite of seven Discovery and L’Atalante sediment samples where nematodes were determined to genus (Fig. 7). The genus *Daptonema*, which was present in all but one sample, dominated most of these sediment samples. The nematodes encountered in the lower halocline samples could not be assigned to a known genus as they were too degraded. Nematodes were not identified to genus in Urania samples.

**Fig. 8:**
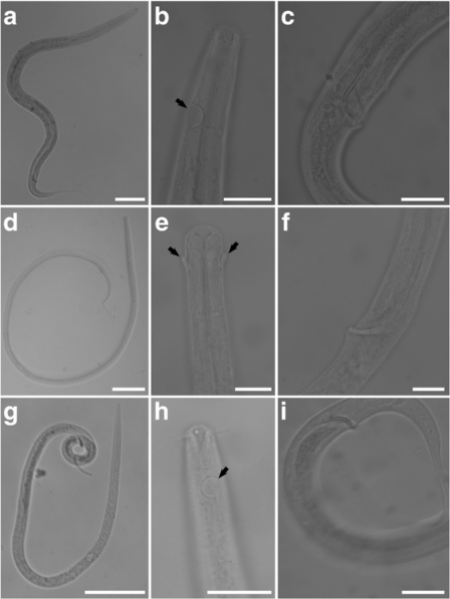
Examples of micrographs of *Daptonema* nematodes obtained from sediment samples. **a**–**c** Morphotype 1 (L’Atalante normoxic normal saline, 611c3). **a** General habitus of Morphotype 1. **b** Section showing head and one amphid (arrow) of Morphotype 1. **c** Spicule of Morphotype 1. **d**–**f** Morphotype 2 (Discovery mid-halocline, 610c16). **d** General habitus of Morphotype 2. **e** Section showing head of Morphotype 2 and the two amphids (arrows). **f** Spicule of Morphotype 2. **g**–**i** Morphotype 3 (L’Atalante Upper Halocline, 611c5). **g** General habitus of Morphotype 3. **h** Section showing head and one amphid (arrow) of Morphotype 3. **i** Spicule of Morphotype 3. Scales: **a**, **d**, **g** = 100 μm; **b**, **c**, **h**, **i** = 20 μm; **e**, **f** = 10 μm

Examination of the male *Daptonema* specimens allowed morphospecies assignments. Interestingly, three *Daptonema* morphospecies were encountered (Fig. 8): one occurred exclusively in the two normoxic, normal saline control samples (611 c3, 610 c11), a second *Daptonema* morphotype was present in the Discovery mid-halocline sample (610 c16), and a third *Daptonema* morphotype was present in the L’Atalante upper halocline sample (611 c5). For each of these morphospecies, all males present in the noted sample(s) were of that morphotype, suggesting the possibility of endemic species in the two haloclines examined. The number of males in each sample was 7 in 611c3, 1 in 610c11, 5 in 610c16, and 33 in 611c5.

### Quantification of nematodes

The highest nematode abundances were observed in Urania normoxic, normal saline control samples collected in the normoxic-halocline transition, immediately adjacent to the upper halocline (>3800 indiv. 10 cm^−2^; Table 3; core 607-608cC in Fig. 1c). Normoxic control samples collected further upslope from the Urania halocline had very few nematodes in the materials we processed (0–31 indiv. 10 cm^−2^). Normoxic control samples of Discovery had patchy distributions, sometimes with high densities (1938 indiv. 10 cm^−2^) and others with low densities (<20 indiv. 10 cm^−2^). Discovery upper haloclines had similarly patchy distributions, with as many as 1119 indiv. 10 cm^−2^ and as few as ~42 indiv. 10 cm^−2^.

**Table 3:** Mean nematode densities and refractometer data (salinity), presented by core designation (sample ID), per deep-sea hypersaline anoxic basins (DHAB) and habitat. Additional file 8 presents density data from each aliquot

DHAB	Habitat	Salinity	Sample ID	Number of aliquots	Number/10 cm^2^	Std dev	Minimum in situ volume processed (cm^3^)
Urania							
	Normoxic, normal saline control	NA	608-3	2	31.25	44.19	4.2
	Normoxic, normal saline control	NA	607-4	2	0	0	4.8
	Normoxic control/halocline transition	NA	607-8	2	3878.79	2228.46	0.7
	Normoxic control/halocline transition	NA	607-8C	2	594.49	128.06	1.3
	Upper halocline	NA	607-10	4	11.08	22.15	3.8
	Mid-halocline	NA	608-11A	4	26.7	31.71	3.8
	Lower halocline	NA	607-8 F	1	0	NA	1.4
	Lower halocline	NA	607-8E	1	7.04	NA	12.6
							32.5
Discovery							
	Normoxic, normal saline control	NA	609-6	3	1937.5	225.35	0.5
	Normoxic, normal saline control	50	610-11	4	16.63	30.64	16
	Upper halocline	NA	609-7	3	41.67	72.17	2.7
	Upper halocline	85	610-14	3	1119.05	802.84	0.4
	Mid-halocline	102	610-16	1	7.32	NA	23.2
	Mid-halocline	102	609-10 H	1	0	NA	1.7
	Mid-halocline	116	610-13	1	17.3	NA	9.3
	Mid-halocline	130	610-3	2	0	0	0.3
	Mid- to lower halocline	NA	609-1	2	0	0	1.6
	Lower halocline	NA	609-14	4	1.45	2.89	20.5
	Lower halocline	NA	610-9	3	0	0	2.2
	Lower halocline	NA	609-4	entire	0	NA	31.7
	Lower halocline	146	610-4	1	1.17	NA	17.1
							127.1
L’Atalante							
	Normoxic, normal saline control	41	611-3	3	76.55	94.25	16.5
	Normoxic, normal saline control	41	611-8	2	62.5	0	0.3
	Upper halocline	44	611-5	3	123.53	57.16	17.3
	Upper halocline	45	611-C	1	246.58	NA	1.1
	Upper halocline	46	611-4	1	181.82	NA	0.6
	Mid-halocline	53	611-10	4	5.47	10.95	3.8
	Mid-halocline	69	611-2	entire	0	NA	31.7
	Lower halocline	190	611-14	3	0	0	1
	Lower halocline	220	611-17	entire	0.5	1	31.7
	Lower halocline	220	611-18	entire	0	NA	31.7
							135.4

Also listed is the number of aliquots analyzed and amount of sediment processed. Processed sediment volumes are summed at the bottom of each DHAB. NA, Not available; Std dev, Standard deviation; ID, Identification

L’Atalante had the overall lowest nematode densities compared to the other DHABs. In L’Atalante, the upper halocline samples had the highest nematode densities (~124-247 indiv. 10 cm^−2^), with L’Atalante normoxic control samples having considerably lower densities (63–77 indiv. 10 cm^−2^). The nematode densities in Urania upper and mid-halocline were also detectable, but always <27 indiv. 10 cm^−2^.

Mid-halocline samples typically had very low nematode abundances and in some cases, nematodes were not detected. In general, nematodes were absent from the deepest halocline samples, although in a few cases some specimens were noted.

### Nematode viability suggested by CellTracker™ Green

**Fig. 9:**
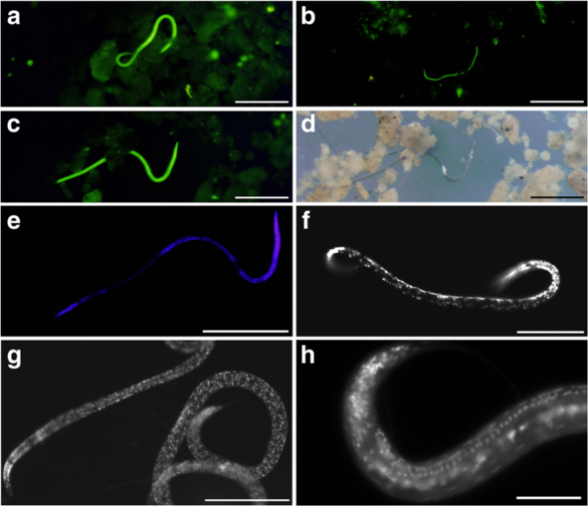
L’Atalante upper halocline nematodes labeled in situ with CellTracker Green (611cC). **a**–**c** Epifluorescence micrographs showing labeled nematodes in sediments (480-nm excitation; 520-nm emission). **d** Paired image of (**c**), in transmitted light. **e**–**h** Epifluorescence images of DAPI staining. **e** Same nematode shown in (**c**, **d**). **f**–**h** Nematodes showing clear DAPI staining of nuclei but no evidence of uniform endo- or ectobionts. Scales: **a**, **c**–**e**, **g** = 250 μm; **b** = 500 μm; **f** = 100 μm; **h** = 50 μm

Sediment cores incubated in situ with CellTracker Green had fluorescently-labeled (i.e. with active esterases) nematodes from some, but not all, samples investigated (Fig. 9a–c; cores with letter designations in Table 3). Five core-top samples were examined for CellTracker Green-labeled nematodes: one L’Atalante upper halocline pushcore, one Discovery mid-halocline pushcore, two Urania lower halocline pushcores, and one normoxic control pushcore collected adjacent to Urania. The three samples that had CellTracker Green-labeled nematodes were the Urania normoxic control, L’Atalante upper halocline, and one of the two Urania lower halocline cores. The other two cores did not reveal CellTracker Green-labeled nematodes in the aliquots examined.

A comparison of nematode viability in sieved sediments using transmitted light versus epifluorescence optics for CellTracker Green shows the distinct labeling of CellTracker Green (Fig. 9c, d). Once specimens were isolated, for those specimens that were incubated with DAPI, the DAPI signal was clear in the nuclei (Fig. 9e–h). DAPI staining did not reveal evidence of nematode epibionts or endobionts. Unfortunately, only one in situ CellTracker Green core was recovered from L’Atalante; the others failed upon collection due to a valve malfunction.

Loriciferans were not detected in any of our samples incubated in situ with CellTracker Green.

### Nematode viability suggested by ultrastructure

Nematode specimens (n = 86) isolated from multiple samples from all three DHABs were processed for ultrastructural examination. A sufficient number of specimens for examination of cellular ultrastructure and assessment of viability, as well as for a survey for possible adaptations such as presence of symbionts, was only obtained from L’Atalante (n = 7). All specimens from Discovery and Urania samples were lost during processing (i.e. solution changes).

**Fig. 10:**
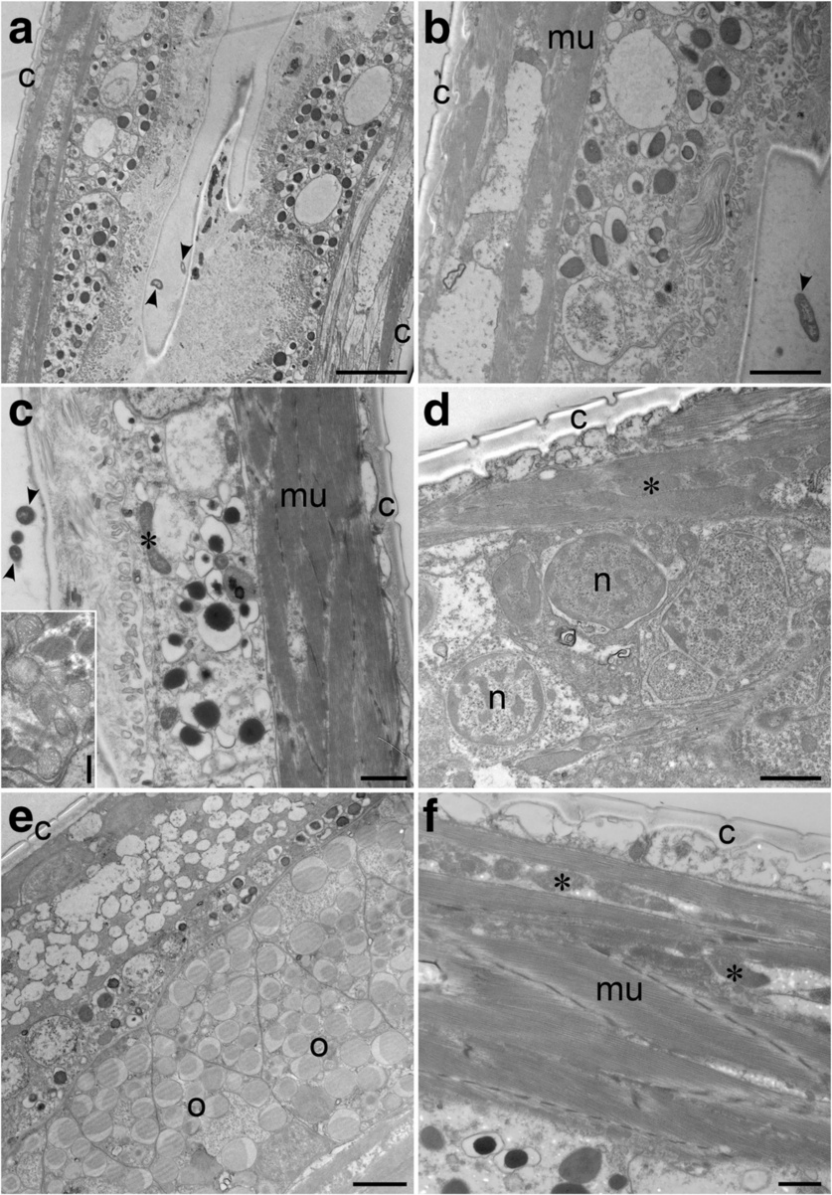
Transmission electron micrographs showing longitudinal sections of three nematodes from L’Atalante normoxia control sediments (611c3). **a**–**c** Portions of gut with ingested bacteria (arrowheads). Inset in c: Mitochondria in same specimen. **d** Nuclei and muscle cells. **e** Portion showing cells, potentially oocytes, with copious lipid. **f** Muscle cells and mitochondria. c, Cuticle; *, Mitochondria; mu, Muscle; n, Nuclei; l, Lipids. **a**–**c**, **e** = specimen 1; **d** = specimen 2; **f** = specimen 3. Scales: **a** = 4 μm; **b**, **e** = 2 μm; **c**, **d**, **f** = 1 μm; inset = 500 nm

**Fig. 11:**
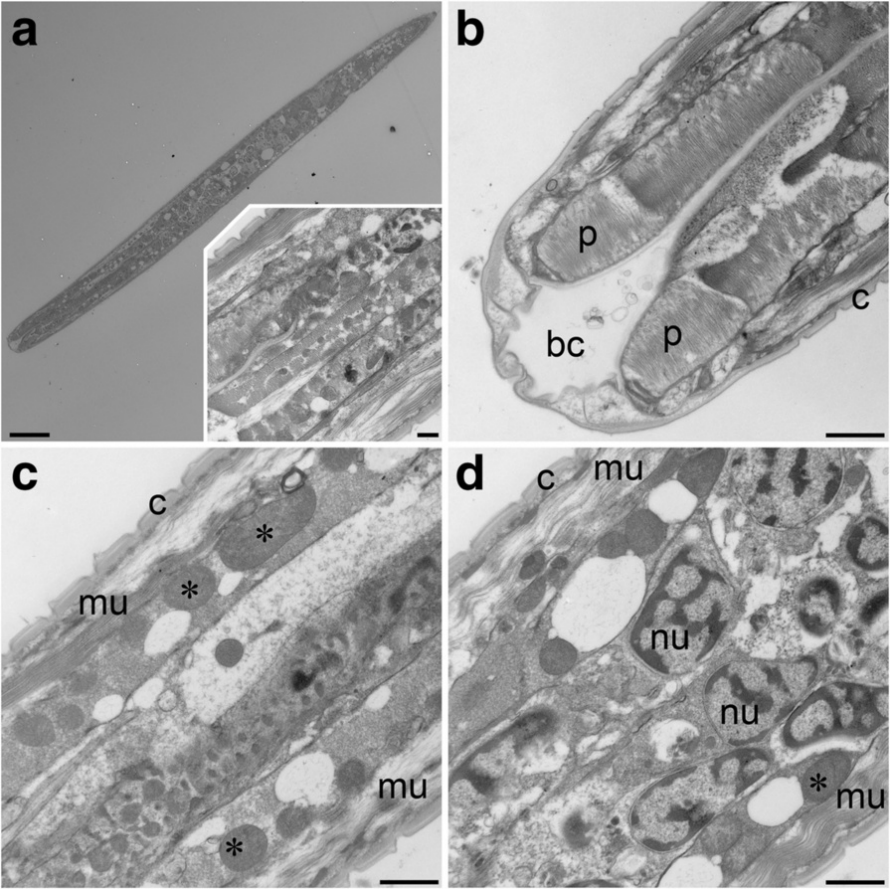
Transmission electron micrographs showing longitudinal sections of nematode from L’Atalante upper halocline (611c5). **a** Overview. Inset: Higher magnification overview, posterior of mouth. **b** Image of anterior end showing buccal cavity (b) and procorpus (p). **c** Mitochondria (*), muscle tissues (mu) and cuticle (c). **d** Cuticle, muscle tissue and nuclei (n) with condensed cromatin. Scales **a** = 10 μm; **b**–**d** = 1 μm; inset = 500 nm

The ultrastructure of nematodes (n = 4) from the normoxic control sediments adjacent to L’Atalante was well preserved and identifiable (Fig. 10). Mitochondria, nuclei, and muscle tissue were all readily identified in these specimens, as were bacteria in the gut. The ultrastructure of one nematode specimen from L’Atalante upper halocline also had identifiable mitochondria, nuclei and muscle tissue (Fig. 11). In addition, the buccal cavity and pharynx of this specimen were observed (Fig. 11b). Closer examination reveals that the muscle tissues in the specimen from the upper halocline appear different from those of the specimens from the normoxic control sediments (compare Fig. 11b–d to Fig. 10c, d, f). It is possible that this upper-halocline specimen was not living at the time of fixation. A second specimen from the L’Atalante upper halocline did not section properly and, thus, could not be examined with TEM.

**Fig. 12:**
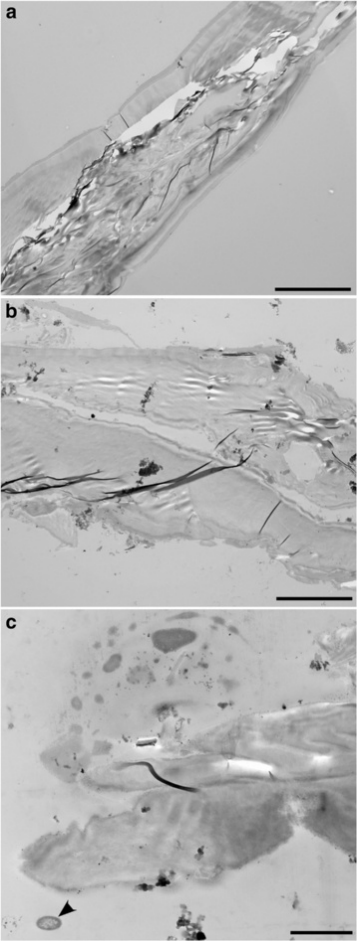
Transmission electron micrographs showing longitudinal sections of suspected nematode remnants from L’Atalante lower halocline (611c17). **a** Specimen 1 showing suspected nematode cuticle and degraded muscle tissue. **b** Specimen 2 showing remnants of suspected gut and cuticle. **c** Specimen 2 showing possible anterior end. Note well preserved prokaryote (arrowhead) in lower left. Scales: **a**, **b** = 10 μm; **c** = 2 μm

The two items examined from the L’Atalante lower halocline appeared to be comprised of highly degraded organic material, perhaps of nematode origin (Fig. 12). What appears to be the cuticle of one specimen remained visible (Fig. 12a). What may have been the gut was visible in the second specimen (Fig. 12b). To show that the fixation of this lower halocline material was adequate, it is worth noting that a well-preserved prokaryote is visible adjacent to one of the specimens (Fig. 12c).

### Prokaryote associates of nematodes

Phase contrast, differential interference contrast (DIC), and epifluorescence (DAPI) images can often reveal if epibionts are present on nematode cuticles (e.g. [30, 31]). TEM images are expected to show epibionts (e.g. [32]) as well as endobionts; the only obvious prokaryotes observed in TEM were those in the nematode digestive tract or as loosely associated materials (Fig. 10a–c). Thus, we saw no evidence for putative symbionts or bacterial associates of the nematodes.

### Bryozoan abundance and viability

**Fig. 13:**
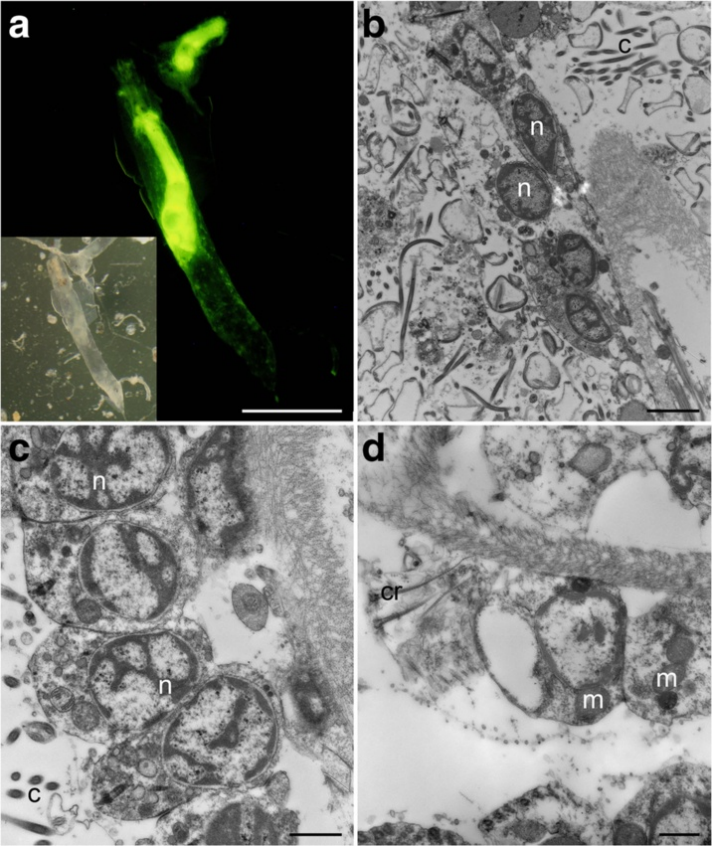
Living *Ctenostomata* sp. (Bryozoa) (Urania normoxic, normal saline/halocline transition; 607-608cC). **a** Two specimens labeled with CellTracker Green demonstrating esterase activity. **b**–**d** Transmission electron micrographs showing intact organelles such as nuclei (n), mitochondria (m), cilia (c), and ciliary root (cr). Scales: **a** = 400 μm; **b** = 2 μm; **c**, **d** = 1 μm

Bryozoans in the Urania transition between normoxic, normal saline and upper halocline sample (607-608cC) labeled with CellTracker Green (Fig. 13a). Two aliquots produced estimated abundances of 260 and 244 indiv. 10 cm^−2^, for a mean abundance of 252 indiv. 10 cm^−2^. The ultrastructure of three of those bryozoans revealed that all three specimens appeared to be live at the time of fixation. Specimens had intact nuclei, mitochondria, cilia, and other organelles (e.g. muscle; Fig. 13b–d).

## Discussion

Metazoans were present in many, but not all, of our DHAB sediment samples. It is important to note, however, that presence does not necessarily equate to viability. Data from our various analyses can inform on the health and activity of the metazoan communities in these DHAB environments; these discussions are presented below by higher taxonomic group.

The physicochemical characteristics of the DHAB haloclines are important to consider when evaluating the likelihood of viability for metazoan taxa. As noted, oxygen was sometimes detectable in the few surface millimeters of halocline sediments [21]. The surface 1-mm of lower halocline sediments were more depleted in oxygen compared to upper halocline samples (typically <10 μM O_2_ vs. 10–70 μM O_2_, respectively). In general, lower halocline sediments are expected to be anoxic while upper halocline sediments are more likely to have some dissolved oxygen. It may be relevant to note that oxygen concentration can be increased during sample handling, so detection of [O_2_] in a recovered pushcore from the lower halocline does not necessarily equate to detectable [O_2_] in situ. Furthermore, and as noted previously, the brine chemistries differ greatly from seawater as well as between the three DHABs (Table 1; [13]). For example, sodium concentrations vary significantly between typical seawater and L’Atalante, Discovery, and Urania brines (528, 4674, 68, 3503 mM, respectively), as do potassium (11, 369, 20, 122 mM, respectively) and sulfide (2.6 × 10^−6^, 2.9, 1, 16 mM, respectively) concentrations. Also, the density of all the brines is substantially greater than that of typical seawater. Thus, it may not be surprising to note the presence of anthropogenic items floating at the brine interface (e.g. Fig. 1a). Such a density gradient may be relevant when contemplating biological attributes of communities across these interfaces. Connectivity between the water column above these DHABs and the brines (and associated sediments) may thus be minimal, with halocline sediment habitats experiencing varied connectivity with the upper water column depending on position within the halocline.

### Crustaceans

Because it is generally agreed that harpacticoid copepods, which are mostly benthic, do not tolerate oxygen depletion or anoxia for considerable periods of time (e.g. [33–35]), it is not surprising that all copepods microscopically observed from halocline samples were either empty exoskeletons or carcasses with little or degraded tissue (Fig. 2), nor is it surprising that harpacticoid copepod rRNA sequences were absent in our dataset since recovery of intact RNA from dead or inactive organisms is less likely than recovery from active organisms (Table 2). The presence of remnant or degraded harpacticoids in halocline samples can be explained because they may have been transported downslope by benthic storms [36, 37] or other disturbances such as turbidity flows and slumps [36]. Although we are not aware of documented benthic storm occurrences in the vicinity of the DHABs, benthic storms have been noted in the Mediterranean [38]. Further, some deep-sea harpacticoids are known to emerge from sediments upon occasion (e.g. [39]), whereby they could be transported via more typical deep-sea currents, into the DHAB haloclines and brines.

A recent study of a shrimp from anchialine caves indicates that some crustaceans can tolerate anoxia for at least 1 week [40]. The most convincing case for a living crustacean population in these DHAB halocline sediments regards cyclopoid copepods. One cyclopoid copepod from the lower halocline of Discovery partially labeled with DAPI (Fig. 2c) and a cyclopoid (*Oithona*) sequence were obtained from the L’Atalante upper halocline. Furthermore, small shrimp-like metazoa of unknown identity were visible via the ROV cameras when the ROV was positioned within the haloclines of all three basins (data not shown). Although it was not clear how long these organisms remained in the halocline, we speculate that they may be at least feeding within the halocline. Thus, while it is possible that copepods in DHAB halocline sediments inhabit that environment, it has yet to be demonstrated that crustaceans can complete a life cycle in anoxia. Conversely, the cyclopoid that was partially labeled with DAPI could have been a dead specimen invaded by prokaryotes or protistan scavengers, which would have also labeled with DAPI.

Our sequence data indicate the relatively common presence of planktonic (calanoid) copepods in our sediments (Table 2). It is likely that adult and/or juvenile calanoid copepods settle from the water column into these halocline sediments. The apparent persistence of amplifiable ribosomal RNA after death has been documented in low oxygen/anoxic (e.g. [41, 42]) and hypersaline [43] environments. Alternatively, it has been shown that encysted embryos of brine shrimp *Artemia franciscana* can tolerate anoxia for considerable periods, up to years [44]; encysted crustacean embryos could be the source of crustacean rRNA sequences in the halocline sediments. Finally, it is possible that upper halocline sediments may support some microaerophilic crustaceans.

In sum, our data indicate that, while it is possible that the copepods detected in these DHABs are anaerobes adapted to the very high salt content of these brines, that possibility is not compelling without additional data.

### Bryozoans

The observation of a bryozoan morphotype in one of our Urania samples suggests patchy distributions of this ctenostome taxon. The significance of a rather abundant (~250 indiv. 10 cm^−2^) occurrence of this bryozoan in a single core is not clear. While it is possible specimens existed in other samples yet were overlooked, it is also possible that the halocline transition provided a favorable environment for these meiofauna. Ctenostome bryozans are known to occur in the bathyal zone as well as in the abyss (reviewed in [29]), but abyssal bryozoan communities remain poorly known.

### Nematodes

Of all the metazoan phyla, nematodes are arguably the most likely to inhabit anoxic DHAB halocline sediments for the following four reasons: (1) nematode presence was already noted in L’Atalante DHAB sediments, although all were reportedly carcasses [1]. (2) A motile nematode has been reported from the sulfidic anoxic zone of the Black Sea [45]. (3) Some nematodes are known for their long-term survival in anoxic, sulfidic habitats [4, 46, 47], with juveniles of some species appearing to be adapted to these conditions [48]. (4) Some nematodes can apparently still reproduce when exposed to anoxic sulfidic conditions [46].

Indeed, we observed dozens of nematodes from many, but not all, samples. The abundances in our normoxic normal saline controls were ~17–77 indiv. 10 cm^−2^, with the exception of nearly 2000 nematodes 10 cm^−2^ in one Discovery normoxic control sample and an absence in a Urania control sample. Nematode densities in other areas of the deep Mediterranean are approximately similar to our normoxic control abundances, with means of 109.8 indiv. 10 cm^−2^ at a 3000-m deep station in the Algero-Provençal, 19.5 indiv. 10 cm^−2^ at a 3000-m deep station in the Ionian Sea, and 61.8 indiv. 10 cm^−2^ at a 3000-m deep station in the Levantine basin [49]. Because our very high nematode abundances (~1940 indiv. 10 cm^−2^ in Discovery normoxic control; 3879 and 595 indiv. 10 cm^−2^ in the Urania halocline transition) were much higher than those of other deep-sea sites, ranging in depth from about 1687–3000 m in the Mediterranean [49, 50] and North-East Atlantic [51], we can consider the DHAB peripheries to be ‘hotspots’ in terms of nematode densities.

In sediment samples incubated in situ with the viability indicator CellTracker Green, we observed labelled nematodes, indicating esterase activity at the time of incubation. Analysis of DAPI-labeled nematodes indicated the expected presence of nuclei throughout many specimens yet lack of copious ectobionts. Because there was no evidence for epibionts or endobionts in DAPI preparations and TEM images, we infer that the positive CellTracker Green labeling reflected nematode viability, not parasitic or scavenging prokaryotes in the body or attached to the nematode cuticle. Ultrastructural analysis revealed the presence of healthy tissue in nematodes from the L’Atalante normoxic control sample and identifiable tissues in a nematode from L’Atalante upper halocline, but no identifiable tissues in specimens recovered from the lower halocline.

Given the above rationale regarding nematode physiological ecology and the general synopsis of our nematode results, it is therefore rather surprising that there were no rRNA sequences of nematodes in our dataset – not from halocline samples or from the normoxic, normal saline control sample. This absence of nematode sequences is even more surprising given the relative abundance of pelagic copepod sequences that were obtained. The PCR primers used in this study are not known to bias against nematodes, although this possibility cannot be excluded for nematodes from such extreme habitats that may be genetically distinct. The presence of the binding sites for the primers used for all the (almost) full-length sequences of *Daptonema*-related (8 *Daptonema* sp. and one *Zygonemella* sp.) taxa found in Genbank suggests, however, that primer bias is likely not an issue. Other possible explanations for the lack of nematode sequences are that, (1) although we used a larger (8 g) than usual (~0.5–1 g) sample of sediment for RNA extraction, this amount may be insufficient due to patchy distribution of active nematodes in any sample extracted or (2) the nematode cuticle does not protect the nematode postmortem as well as the copepod exoskeleton. Additionally, signatures of pelagic copepods may have masked those of benthic nematodes merely due to abundance and rate of addition to the sediments. The deep Mediterranean is considered highly oligotrophic [52–55], so other than near/within halocline/oxycline habitats associated with DHABs where abundant microbial activity occurs (e.g. [14]), abundant benthic metazoan communities are not expected, even in normoxic Mediterranean sediments at ~3.5 km depth. In spite of the fact that we did not recover nematode ribosomal RNA signatures from our samples, our ultrastructural analyses argue that at least some of the nematodes detected in halocline samples were viable, as it would be very unlikely to have the same level of excellent preservation as seen in our nematodes from the normoxic L’Atalante control sample (Fig. 9).

Since (1) abundances of nematodes were considerably lower in the deeper halocline samples compared to upper halocline and normoxic control samples (Table 3), (2) the genus composition of the nematode communities varied by sample (Fig. 7), and (3) *Daptonema* species differed among habitats and DHABs (Fig. 8), we argue that the halocline and normoxic, normal saline control nematode populations were active in those environments. We argue against the possible assertion that the nematodes from all samples were dead.

Given that no identifiable nematode tissue was observed with TEM in either of the nematodes from the lower halocline samples (n = 2, L’Atalante) and since there were very low abundances of nematodes recovered from all additional lower halocline samples (Table 3), we suggest that populations of active nematodes may be limited to the upper haloclines of the DHABs. By inference, we do not expect active nematode populations in the DHAB brines.

### Loriciferans

Individual loriciferans belonging to the same three genera recorded by Danovaro et al. [1] were obtained from our samples. Our specimens very likely represent the same species as those of Danovaro et al. [1], including *Spinoloricus cinziae*, the only one described so far [28]. The positive species identification of our specimens relies on the agreement of the general appearance and, especially, on the presence of the additional spines on the lorica (Fig. 4), a trait that is unique to this taxon [28]. With regard to *Rugiloricus* sp. and *Pliciloricus* sp., it is worth noting that a definitive match between our specimens and those reported by Danovaro et al. [1] cannot be invoked because a single light level photomicrograph of each taxon was provided by those authors, preventing in-depth comparisons. However, the occurrence in the same peculiar habitat should provide some confidence about the taxonomic correspondence between the two loriciferan groups. Our specimens had their lorica composed of numerous longitudinal elements called plicae, a trait that characterizes the *Pliciloricidae*. This family currently includes two genera, *Pliciloricus* and *Rugiloricus*; we used the number of plicae to affiliate our specimens to either of the two genera: about 22 plicae = *Pliciloricus*; 30–60 plicae = *Rugiloricus* [56]. Further, the specimens with the lower number of plicae (Fig. 5c, d) had a very long mouth cone, a characteristic already reported in *Pliciloricus* (i.e. *P. gracilis* from the U.S. Atlantic coast [57]) but unreported for *Rugiloricus.*

Evidence for living loriciferans in the brine of L’Atalante DHAB included uptake of the viability indicator CellTracker Green, which labels active esterases, ^3^H-leucine by specimens in sediment incubations, and cellular ultrastructure analyses that show putative hydrogenosomes, which are specialized organelles heretofore unique to unicellular anaerobic eukaryotes (e.g. [58]), and endobionts but lack of easily identifiable mitochondria [1]. In addition, an unspecified number of loriciferans were observed to have a structure resembling an oocyte, causing the authors to assert that the brine population was fecund, and a number of postlarva exuviae were found, leading to the belief that metamorphosis of postlarva to adult occurs in the brine.

While the assertions of Danovaro et al. [1] are exciting, there are other potential explanations besides the conclusion that the DHAB loriciferans are true anaerobes. With regard to the evidence based on staining, radiolabeling and ultrastructure, one alternative scenario is that the loriciferan tissues within their lorica (exoskeleton) were actually dead, degraded to varying degrees and inhabited by living anaerobic bacteria, archaea, and/or fungi. This possibility is suggested for several reasons. First, prokaryotes within degrading tissue could account for the positive rose Bengal staining within the loriciferan exoskeleton (lorica), as has been shown for foraminifera [59]. Second, although the loriciferans from anoxic sediments incorporated radiolabeled leucine, bacteria are also known to uptake leucine (e.g. [60, 61]); indeed, even halophilic archaea have been shown to uptake leucine [62]. Third, although esterase activity was reported via positive CellTracker Green labeling in the loriciferans, bacteria also label with CellTracker Green [10] and could cause the loriciferans to fluoresce. Fourth, although ‘hydrogenosome-like organelles’ were reported in loriciferans, it is possible that these structures are actually prokaryotes, as there are few morphological traits specific to hydrogenosomes [63]. Indeed, the only intact cytoplasmic features shown in Danovaro et al. [1] besides apparent hydrogenosomes were ‘possible endosymbiotic prokaryotes’ (Figure 4d–f in ref [1]), which could be scavengers or parasites. Even if the structures were bona fide hydrogenosomes, the time it takes for those organelles to degrade after death is unknown; it is possible that hydrogenosomes do not quickly show visible signs of degradation as interpreted for other organelles [59]. As for the alleged evidence for breeding and molting of Loricifera within the basin, an alternative explanation is that the reducing conditions of the permanently anoxic sediments can preserve, for a long time, tissues and organs of dead organisms to the point that the oocytes of some specimens may erroneously appear healthy under light microscopy. With regard to the presence of postlarvae exuvia, they could have reached the brine like any other specimen or remains (i.e. via transport).

Unfortunately, we did not obtain sufficient numbers of loriciferans to perform a complete suite of analyses on this intriguing taxon, including confirmation of intact and identifiable eukaryotic organs and organelles. In addition, it is noted that no rRNA sequences were obtained for loriciferans. We did document, however, that 15 loriciferans were obtained from lower halocline samples of both L’Atalante and Discovery after examining approximately 42 and 16 cm^3^ of sediment (in situ volume), respectively. Importantly, one loriciferan specimen (*Spinoloricus cinziae*) was also obtained from a L’Atalante normoxic, normal saline control sample.

We saw some evidence of potentially intact loriciferan tissue in some of the recovered specimens, where two Rose Bengal stained specimens each had a possible oocyte (Fig. 5b, Additional file 6: Figure S6). It is established for other meiofauna, however, that Rose Bengal staining is not necessarily indicative of viability (e.g. [59, 64]).

It may be that the cuticle of loriciferans may further retard the degradation of soft tissue in this taxon. The minimal DAPI staining may indicate inability of the stain to pass through the lorica or the lorica may slow down bacterial invasion. It is important to note that no other identifiable organs other than putative oocytes were observed in any of the loriciferans examined at adequate magnification (n = 4), and an ultrastructural survey would have been necessary to establish that the oocytes in our specimens were healthy.

Although we found specimens of *Rugiloricus* sp. in both the Discovery and L’Atalante lower haloclines, the likelihood that the same species of any metazoan inhabits both the athalassohaline Discovery lower halocline and the thalassohaline L’Atalante lower halocline is low given the very different water chemistries of those two locales (Table 1). For example, Discovery brine has an extremely high Mg^2+^ concentration (4995 mM), which is approximately an order of magnitude higher than that of L’Atalante (410 mM); L’Atalante brine has more than 65 times more sodium than Discovery (4674 vs. 68 mM); L’Atalante brine has approximately four times more sulfate than Discovery brine (397 vs 96 mM, respectively); and L’Atalante brine has more than six times greater potassium than Discovery (369 vs. 60 mM). Using that same rationale, while we found *Spinoloricus cinziae* in L’Atalante control and L’Atalante lower halocline samples, the likelihood that the same species of *Spinoloricus* inhabits both the normoxic, normal saline control sediments and thalassohaline lower halocline sediments is low due to substantial differences in the chemistry of bottom waters overlying sediments and pore waters.

Because of our observations on loriciferan distribution and the visible state of specimens, we find little evidence for living loriciferan populations in the haloclines of L’Atalante and Discovery. Although we did not examine much material from Urania, that DHAB might be the most hospitable to anaerobic metazoans given the brine of Urania is less saline than the other DHABs [18]. However, the hydrogen sulfide concentration of Urania brine is extremely high (>300 times more than one marine chemocline known to support abundant eukaryotes) [10]. By inference, we await additional definitive data demonstrating loriciferan inhabitation of DHAB lower halocline and brine sediments.

## Conclusions

Our goal for this research was to investigate the presence of living metazoan communities in the DHAB haloclines and brines. Unfortunately, brine samples were not obtained. Our results suggest a community of living nematodes in normoxic, normal saline deep-sea Mediterranean sediments and in the upper halocline portions of several DHABs. There is some evidence suggesting nematode endemicity in the upper to mid-haloclines of L’Atalante and Discovery DHABs. There is also some evidence of enhanced nematode and bryozoan densities in the vicinity of the upper halocline and adjacent normoxic, normal saline regions. Occurrences of nematodes in mid-halocline and lower halocline samples did not provide compelling evidence of a living community. Crustacean occurrences in our samples suggest the possibility of living benthic taxa in the haloclines (e.g. *Pontophilus*), but most crustacean morphotypes and sequences were from planktonic copepod taxa, suggesting an active benthic crustacean community is of low diversity, if it exists in the haloclines. We also strove to confirm the presence of an active loriciferan community in the L’Atalante DHAB, as noted by Danovaro et al. [1]. If we could have contributed stronger evidence of this active community using the different approaches applied, a paradigm shift would be necessary, where textbooks would have to be rewritten to include such metazoan populations completing their life cycle under permanent anoxia. Unfortunately, we could not confirm loriciferan activity due to a paucity of specimens, and the degraded nature of the specimens we did find. A healthy metazoan community in lower haloclines and, by inference, brine zones of DHABs is not supported by our data at this time. A more confident assessment of metazoan fitness and activity can possibly be gleaned from gene expression analysis using reverse transcription quantitative PCR or metatranscriptomics, as recently used to unveil the prokaryotic and whole community activities in the water column of DHABs (e.g. [65, 66]).

## Methods

### Sampling

On RV *Atlantis* cruise AT18-14, sediment samples were collected using the ROV *Jason* from 24 November to 6 December 2011. Samples were collected in and near three DHABS: Urania, Discovery, L’Atalante (see Table 1 for coordinates). All samples were collected with 6.35-cm inner-diameter pushcores designed to prevent contamination during descent and ascent (www.whoi.edu/groups/DSL/). Pushcores were obtained in different zones with respect to where the halocline intersected the seafloor and salinities later confirmed by refractometer. Thus, normoxic, normal saline control samples were collected upslope from the visible manifestation of the halocline impinging the seafloor (Fig. 1; see also Figure 2 in Bernhard et al. [21]). The positive buoyancy of ROV *Jason* prevented sampling the brine proper from any DHAB. When sampling the halocline, the ROV was typically positioned outside (upslope from) the halocline, and reached into the halocline to sample. Minimal disturbance was observed of halocline waters (Schlieren effect) on ROV live video feed. Lower halocline cores were obtained by extending Jason’s manipulator arm into the DHAB as far as possible; when collecting cores underlying the halocline, sampling did not commence until visible disturbance (observable Schlieren) had settled. In the case of sampling sediments under the lower halocline, the conditions made it impossible to view the point at which *Jason*’s manipulator arm contacted the seafloor, resulting in over-penetration of some pushcores. A site was never sampled after ROV re-positioning, which introduces disturbance from the propellers.

We do not feel that living meiofauna escaped sampling due to ROV noise or vibrations because meiobenthic organisms are less than 1 mm long, generally not very fast (e.g. [67]), and typically not hyperbenthic. If there was a response to the ROV, we did not expect the meiofauna to get very far; we expected them to remain within sediments and thus be sampled. Images and video of the sampling can be seen at ROV Jason’s virtual van (http://4dgeo.whoi.edu/webdata/virtualvan/html/VV-at18-14/index.html).

Upon *Jason* recovery aboard support vessel RV *Atlantis*, pushcores were taken within ~5 minutes to the ship’s environmental room, which was set at 9.5 (±0.5 °C), which was ~4 °C lower than in situ temperature (~14 °C). The cooler temperature was to expedite cooling of the samples, which were subject to warming on ascent and ROV recovery. Our goal was to obtain at least three pushcores (biological replicates) of each habitat from each DHAB; one was intended primarily for sequencing, one for quantification and one for imaging. Pushcores intended for sequencing were subcored and directly frozen, as described below. Pushcores for metazoan quantification (counts) and imaging were first sampled for overlying water to estimate salinity via a hand-held refractometer. Because of the high ionic concentrations, up to 10-fold dilution was required to register on the refractometer. The refractometer was not expected to provide precise salinity data, especially for Discovery’s very high MgCl_2_ brine. Rather, the data were intended as a relative indicator between samples from different halocline horizons in Discovery and L’Atalante; refractometer data was not collected for Urania pushcores. Sometimes bottom water was not available due to full penetration of the pushcore barrel; refractometer data are not reported in those cases. Halocline samples are designated in the following descending order by their relative location (based on refractometer reading, visible siting): upper halocline, mid-halocline, and lower halocline. After recording the refractometer reading, the surface centimeter of each pushcore was sectioned and retained. Each of the sediment samples designated for counts and imaging was preserved in either 3 % TEM-grade glutaraldehyde in a 0.1 cacodylic acid sodium salt buffer (pH 7.2) or 3.8 % formaldehyde buffered with phosphate buffered saline. Glutaraldehyde-preserved samples were intended for ultrastructural analyses with TEM (see below) and also imaging with light microscopy; formalin-preserved samples were intended for quantification and/or imaging.

Because pressure and temperature changes during ascent may cause metazoans to die, a subset of pushcores were incubated in situ using CellTracker™ Green (chlorofluoromethyl fluorescein diacetate; Life Technologies), which is reported to label marine meiofauna and microbes [4, 10, 68] as well as L’Atalante loriciferans [1]. CellTracker Green is a viability indicator that relies on active esterases, which are known to function in Urania brine [69]. For these samples, pushcores were partially inserted into the seafloor, injected with CellTracker Green and left to incubate in place for ≥24 hours prior to collection. Two to three days later, during the next ROV dive, these pushcores were removed from the seafloor and brought to the sea surface. Because only one dive was performed in L’Atalante, those incubations were initiated early in the dive and were terminated after recovery later that day, resulting in an incubation of ~12.5 hours, ~7 hours of which were at depth. Injector pushcores were sliced and preserved in glutaraldehyde as noted above. Similar ‘injector’ pushcores were used for related work [21] and previously [70] for seafloor incubations using the same fluorescent probe.

### Characterization of sediment metazoa using SSU rRNA

For isolation of metazoan RNA, select pushcores were subsampled with a sterile 1.4-cm inner diameter syringe corer. These subcores were frozen at −80 °C while at sea. At Woods Hole Oceanographic Institution, the cores were transferred to −20 °C for an hour and then to 4 °C for 10–15 minutes. This allowed the outer part of the subcore sediments to thaw in order to be pushed out of the syringe, but the subcores were still frozen when sectioned. The top 2 cm were placed in a sterile tube and stored frozen (−80 °C) until RNA extraction. RNA extraction, cDNA synthesis, amplification of eukaryotic small subunit rRNA genes, and subsequent analyses were identical to those described in Bernhard et al. [21].

### Metazoan quantification and imaging

Quantitative aliquots of sediment samples were processed for metazoan enumeration and imaging, loriciferan searches, nematode morphotype identifications, and nematode and bryozoan ultrastructural analyses. Initially, all workers were blind to sample origin (i.e. habitat). However, with time, it was obvious to many which samples were from haloclines versus control (aerated) sites, due to differences in sediment color. Because the goal was to obtain as many metazoans as possible, this knowledge did not impact the outcome. Nematode and loriciferan identifications were fully blind. Many technical aliquots (portion of pushcore sample) were sieved over a 63-μm screen and the coarser fraction was examined for individuals. Also, metazoa were isolated from aliquots of sediments along with protozoa [21] using a Percoll^®^ density gradient extraction [71], the supernatant of which was filtered and the blackened filter mounted on microscope slides after labeling with DAPI to distinguish prokaryotic associates (putative symbionts) and/or nuclei. All metazoan taxa observed (and many specimens of each) in these Percoll^®^ preparations, were photographed. Typically, these specimens were imaged with transmitted light (e.g. DIC or phase contrast optics) and also with epifluorescence (377-nm excitation; 447-nm emission) to image DAPI patterns, using an Olympus BX51 microscope and Olympus DP70 camera or a Zeiss AxioImager.M2 equipped with a Zeiss AxioCam MRm digital black and white camera or a Canon EOS Rebel T2i color digital camera. In cases where the metazoan was found on the blackened filter, DIC or phase contrast imaging were not possible. In cases where the sediment sample had been incubated in situ (on the seafloor) with the esterase indicator CellTracker Green (see above), digital epifluorescence images were also taken with 472-nm excitation, 520-nm emission, with either the Olympus BX51 or a Leica MZFLIII dissecting microscope.

The bryozoa and some loriciferans were also imaged using a Nikon Eclips 90i microscope fitted with DIC optics and a DS-5 M Nikon digital camera driven by Nikon ACT-2U software v.1.4.

Because a portion of our samples were sieved using a 63-μm screen, which may be considered large for deep-sea nematode isolation [72], our nematode abundances should be considered minimum rather than absolute densities.

### Nematode morphotype identifications

Aliquots of select aldehyde-preserved samples were sent to the Marine Biology Research Group of Ghent University for nematode morphotype analyses. The >32-μm fraction of sediments was subject to a Ludox^®^ density-gradient centrifugation; the supernatant was stained with Rose Bengal and metazoan meiofauna were sorted under a stereomicroscope and identified to coarse taxonomic level [72]. Nematodes were handpicked and mounted on slides. After, these specimens were examined with a Leica DMR microscope and assigned to genus when possible. Occasionally, areas of interest (e.g. heads, tails) were digitally imaged using the Leica Application Suite software. All males of the most abundant nematode genus were assigned to morphospecies based on morphometric characteristics (e.g. body length, body width, position and size of the amphids). The analyzed samples were from a known volume of sediment, so metazoan densities could be calculated; these data were added to counts obtained via sieving and with Percoll isolations as described above. When loriciferans were encountered in these preparations, images were obtained in some cases.

### Cellular ultrastructure: nematodes and bryozoans

As noted, one-centimeter sections of sediment pushcores were fixed using TEM fixative (3 % glutaraldehyde/0.1 M cacodyllic acid, pH 7.2). In the laboratory, nematode specimens were isolated from the >63-μm sediment fraction and embedded for TEM as per standard protocols (e.g. [32]). Specimens were sectioned (80-nm thick sections; Plant Biology Department, University of Georgia), post-stained in saturated uranyl acetate (50 % in ethanol) and lead stain [73], and examined with a JEM-1210 Transmission Electron Microscope equipped with an Advanced Microscopy Techniques XR41C Bottom-Mount CCD camera or JEOL JEM-1011 (University of Georgia). Specimens were surveyed for cell structure, specifically searching for intact organelles (nuclei, peroxisomes, Golgi), muscle, and prokaryotic associates (commensals, parasites, scavengers [59]; ingested foodstuff). Fluorescent bryozoan specimens were noted in the >63-μm fraction of one injector pushcore sample from the halocline transition of Urania (607-608cC); these were imaged, isolated, and designated for light microscopy or TEM as described above.

## Availability of data and materials

Cruise data is available at the Biological and Chemical Oceanography Data Management Office (BCO-DMO) web site (http://www.bco-dmo.org/project/2177 and http://www.bco-dmo.org/project/2134), and on the ROV *Jason* Virtual Van website (http://4dgeo.whoi.edu/webdata/virtualvan/html/VV-at18-14/index.html). Supplemental images of loriciferans can be found at the Woods Hole Open Access Server (WHOAS) at http://hdl.handle.net/1912/7550. Sequence data is available at https://trace.ddbj.nig.ac.jp/DRASearch/study?acc=SRP049010 and a mapping file showing how to link sequence identifiers with environmental source (basin and habitat) in Additional file 7. Nematode density data is presented for each aliquot in Additional file 8.

## Additional files

Additional file 1: Figure S1. From the supplemental images of loriciferans, which can be found at the Woods Hole Open Access Server (WHOAS) at http://dx.doi.org/10.1575/1912/7550. Differential interference contrast (DIC) and 4’,6-diamidino-2-phenylindole (DAPI) images of a *Spinoloricus cinziae* loriciferan from L’Atalante deep-sea hypersaline anoxic basin control (Core sample ID 611 c3). This specimen is shown in Figs. 3a, b and 4 of the paper. (PPT 2927 kb) Additional file 2: Figure S2. From the supplemental images of loriciferans, which can be found at the Woods Hole Open Access Server (WHOAS) at http://dx.doi.org/10.1575/1912/7550.Differential interference contrast (DIC) and 4’,6-diamidino-2-phenylindole (DAPI) images of a *Spinoloricus cinziae* loriciferan from L’Atalante deep-sea hypersaline anoxic basin, lower halocline (Core sample ID 611 c14). This specimen is shown in Fig. 3c, d of the paper. (PPT 7578 kb) Additional file 3: Figure S3. From the supplemental images of loriciferans, which can be found at the Woods Hole Open Access Server (WHOAS) at http://dx.doi.org/10.1575/1912/7550. Differential interference contrast (DIC) images of a *Rugiloricus* sp. loriciferan from L’Atalante deep-sea hypersaline anoxic basin, lower halocline (Core sample ID 611 c17). This specimen is shown in Fig. 5a, b of the paper. (PPTX 11311 kb) Additional file 4: Figure S4. From the supplemental images of loriciferans, which can be found at the Woods Hole Open Access Server (WHOAS) at http://dx.doi.org/10.1575/1912/7550. Differential interference contrast (DIC) images of a *Pliciloricus* sp. loriciferan from L’Atalante deep-sea hypersaline anoxic basin, lower halocline (Core sample ID 611 c17). This specimen is shown in Fig. 5c, d in the paper. (PPTX 12024 kb) Additional file 5: Figure S5. From the supplemental images of loriciferans, which can be found at the Woods Hole Open Access Server (WHOAS) at http://dx.doi.org/10.1575/1912/7550. Differential interference contrast (DIC) images of a *Pliciloricidae* sp. loriciferan from L’Atalante deep-sea hypersaline anoxic basin, lower halocline (Core sample ID 611 c17). (PPT 2218 kb) Additional file 6: Figure S6. From the supplemental images of loriciferans, which can be found at the Woods Hole Open Access Server (WHOAS) at http://dx.doi.org/10.1575/1912/7550. Differential interference contrast (DIC) images of a *Rugiloricus* sp. loriciferan from Discovery deep-sea hypersaline anoxic basin, lower halocline (Core sample ID 609 c14). (PPT 2893 kb) Additional file 7: Table S1. From the supplemental images of loriciferans, which can be found at the Woods Hole Open Access Server (WHOAS) at http://dx.doi.org/10.1575/1912/7550. Mapping files linking sample IDs (deep-sea hypersaline anoxic basin and habitat) to sequence IDs found at https://trace.ddbj.nig.ac.jp/DRASearch/study?acc=SRP049010. (XLS 6 kb) Additional file 8: Table S2. From the supplemental images of loriciferans, which can be found at the Woods Hole Open Access Server (WHOAS) at http://dx.doi.org/10.1575/1912/7550. Nematode densities for each aliquot presented by core designation, per deep-sea hypersaline anoxic basin and habitat. (DOC 14 kb)

## Abbreviations

DAPI: 4’,6-diamidino-2-phenylindole
DHAB: Deep hypersaline anoxic basin
DIC: Differential interference contrast
OTU: Operational taxonomic unit
ROV: Remotely Operated Vehicle
rRNA: ribosomal RNA
TEM: Transmission Electron Microscopy
