# Supplementary figures and images for: Metazoans of redoxcline sediments in Mediterranean deep-sea hypersaline anoxic basins
Source: BMC Biol. 2015 Dec 10;13:105. doi: 10.1186/s12915-015-0213-6 (PMC4676161; doi:10.1186/s12915-015-0213-6)

## Slide 1
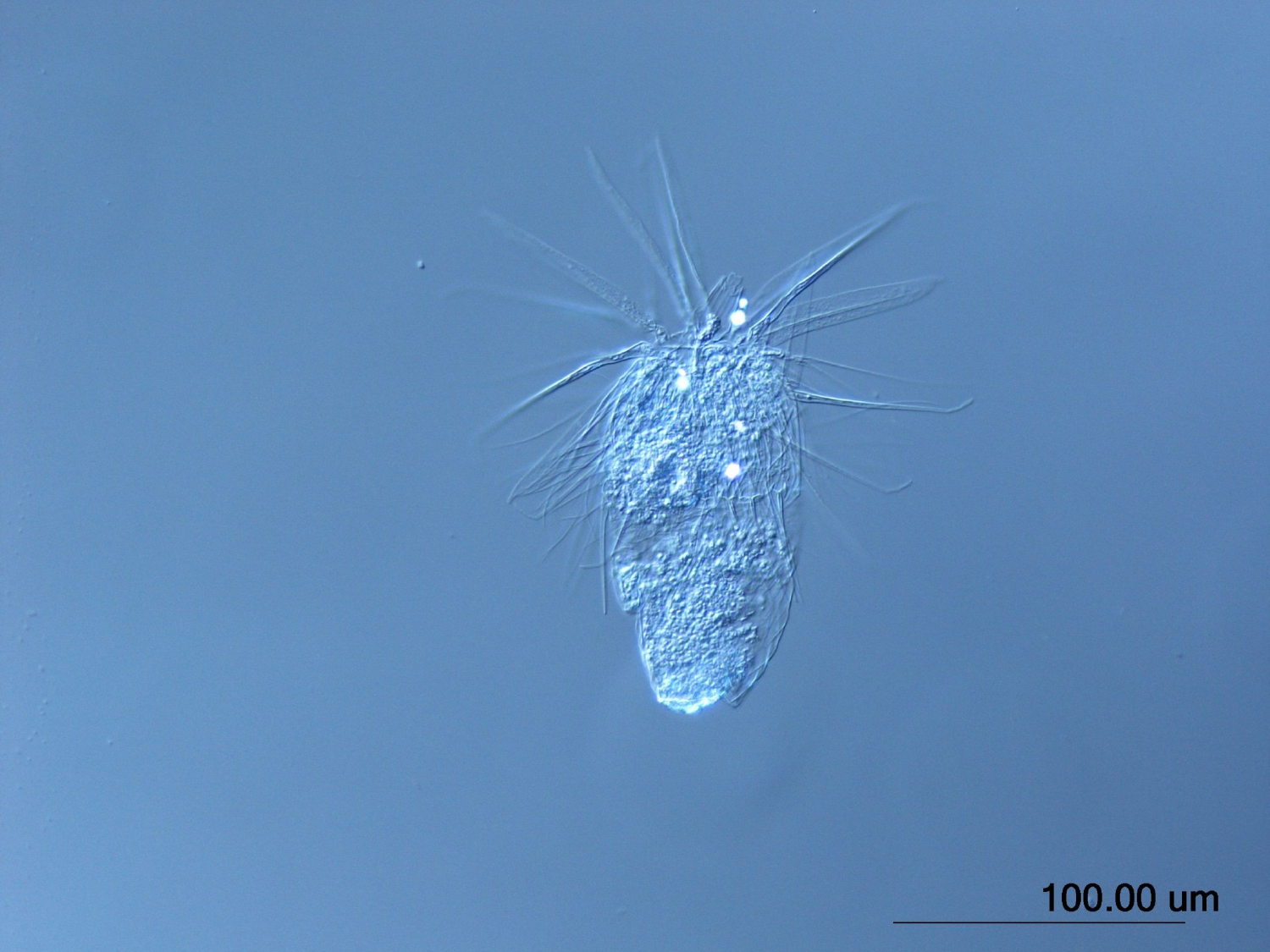

## Slide 2
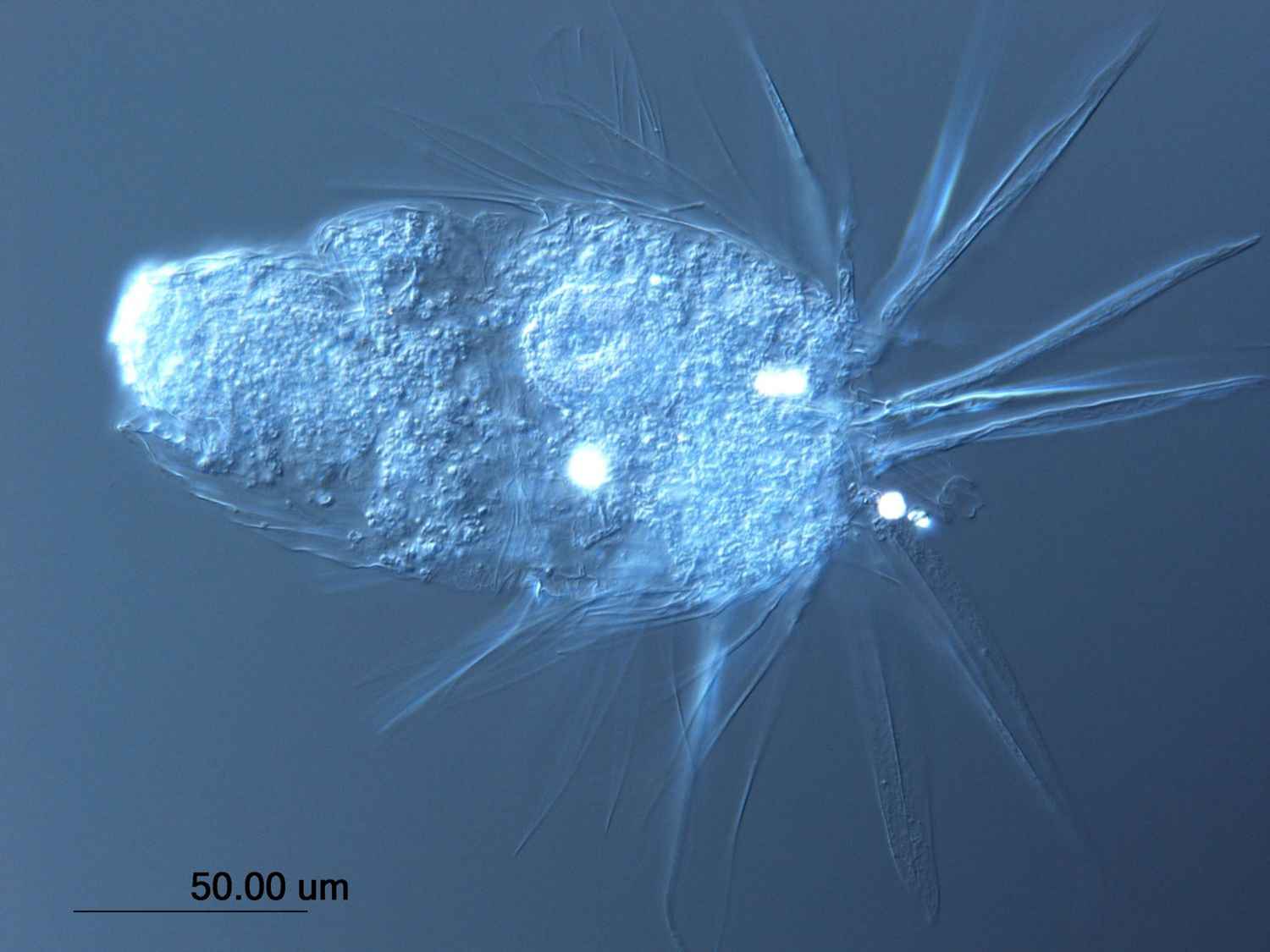

## Slide 3
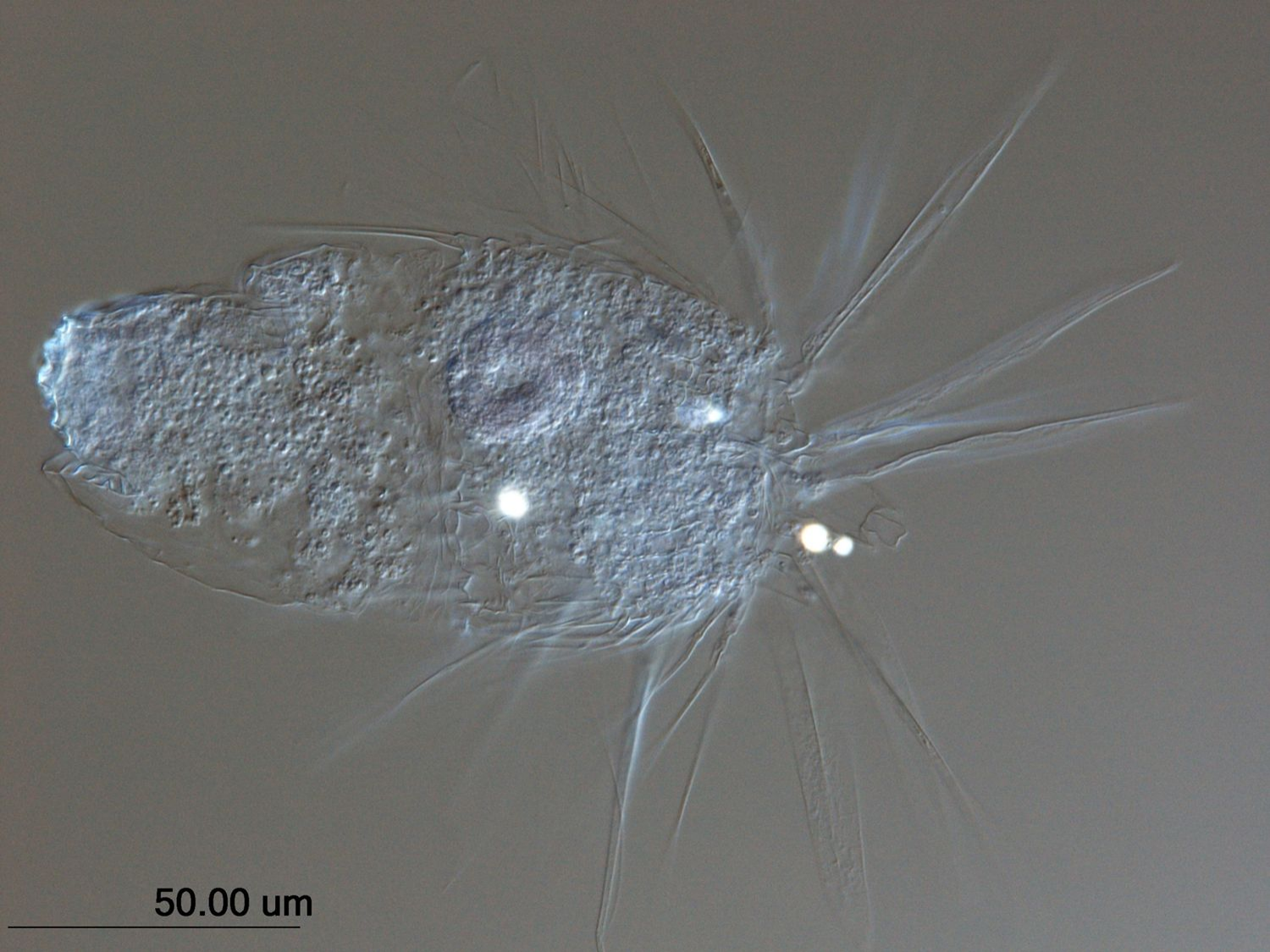

## Slide 4
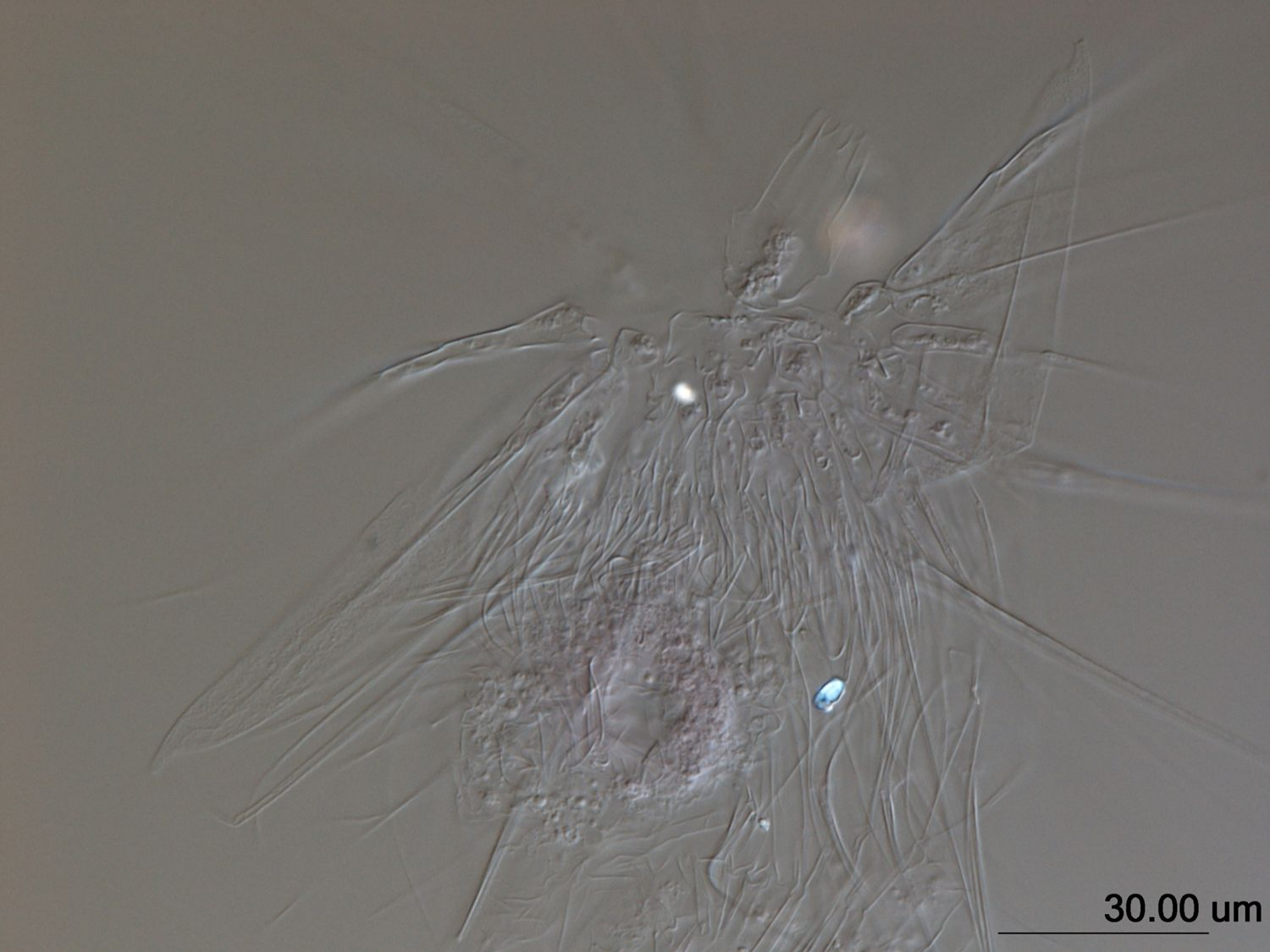

Supplement: Additional file 3: Figure S3. — From the supplemental images of loriciferans, which can be found at the Woods Hole Open Access Server (WHOAS) at http://dx.doi.org/10.1575/1912/7550. Differential interference contrast (DIC) images of a Rugiloricus sp. loriciferan from L’Atalante deep-sea hypersaline anoxic basin, lower halocline (Core sample ID 611 c17). This specimen is shown in Fig. 5a, b of the paper. (PPTX 11311 kb) [file 12915_2015_213_MOESM3_ESM.pptx]

## Slide 1
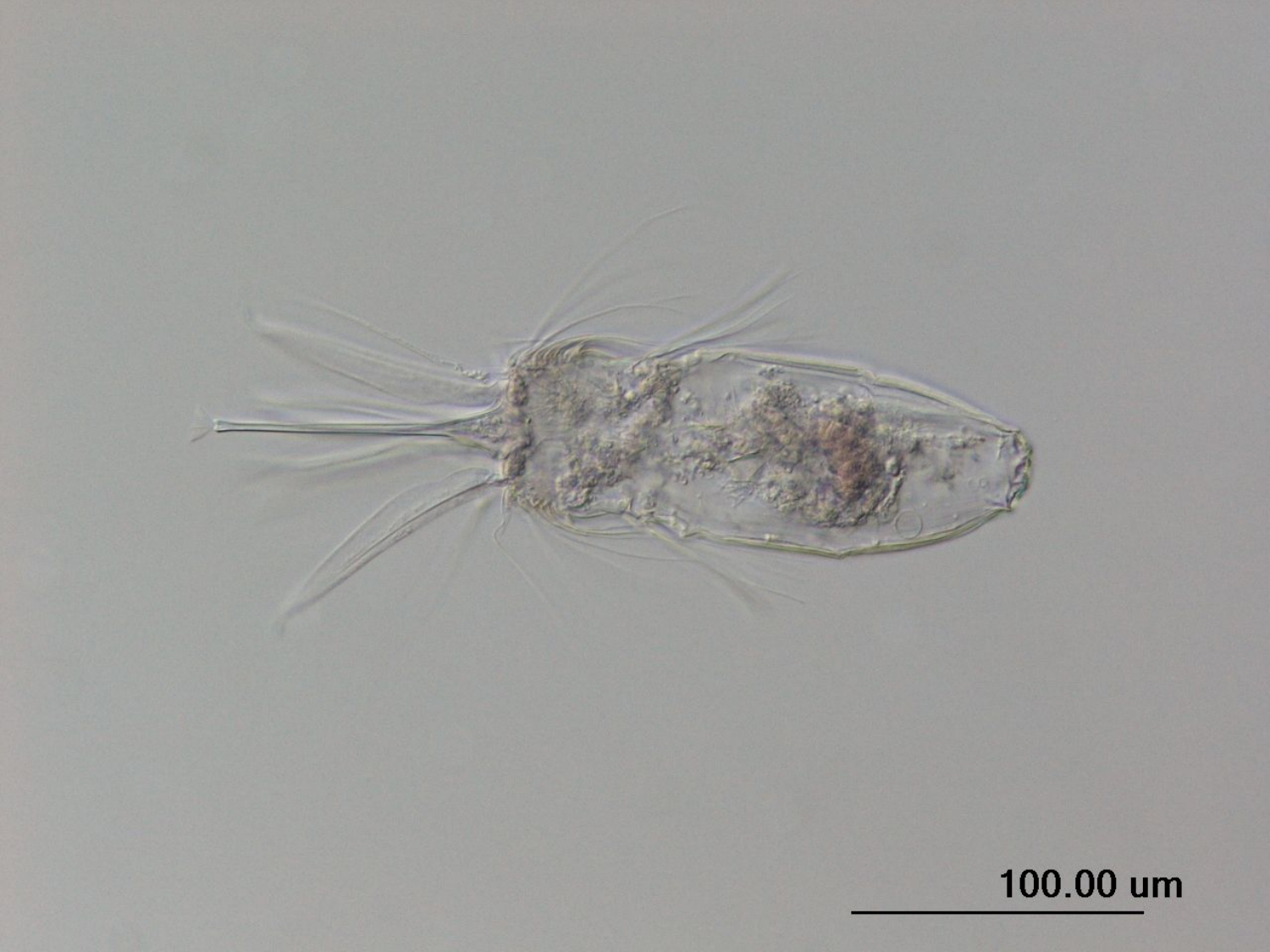

## Slide 2
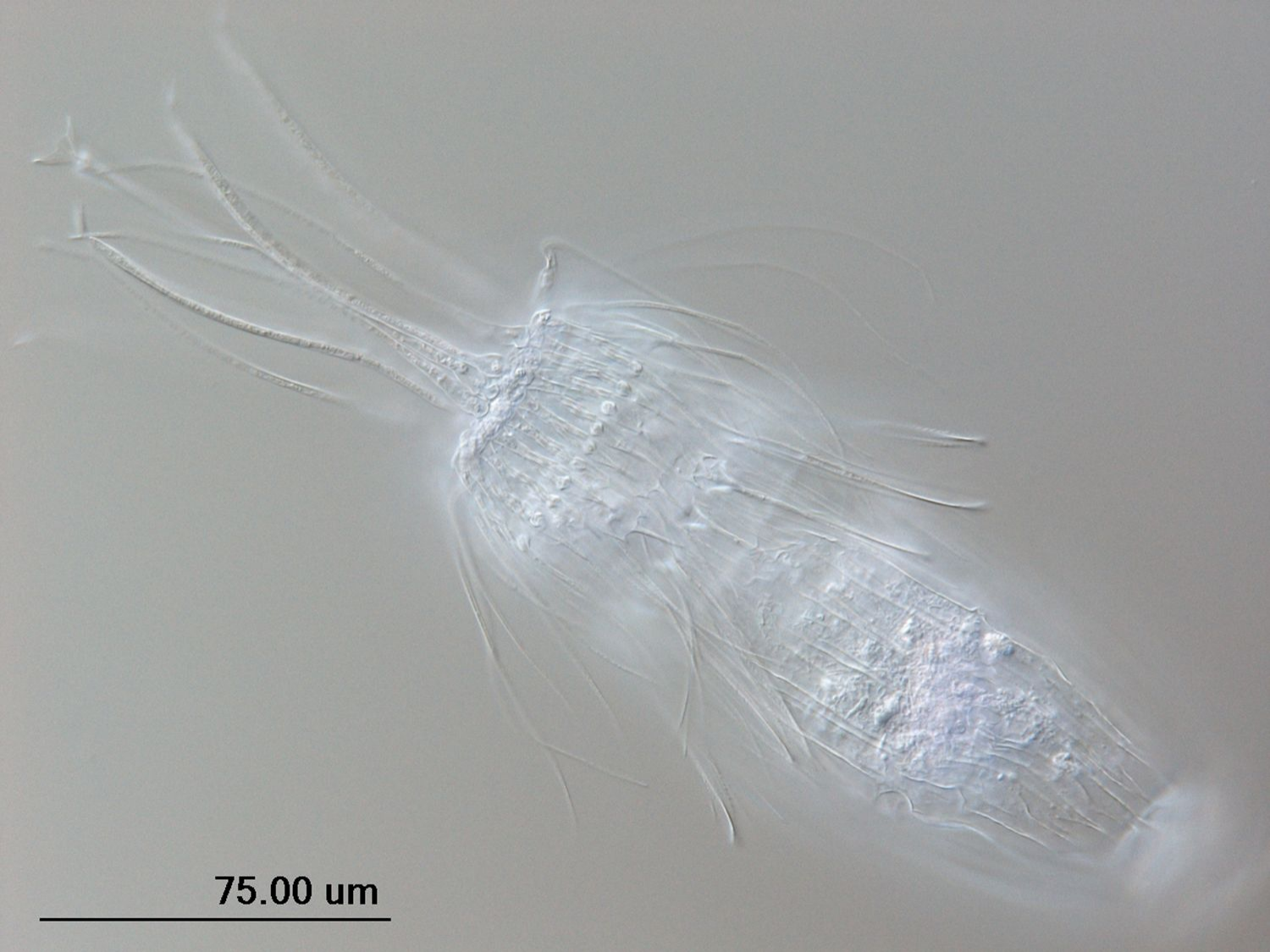

## Slide 3
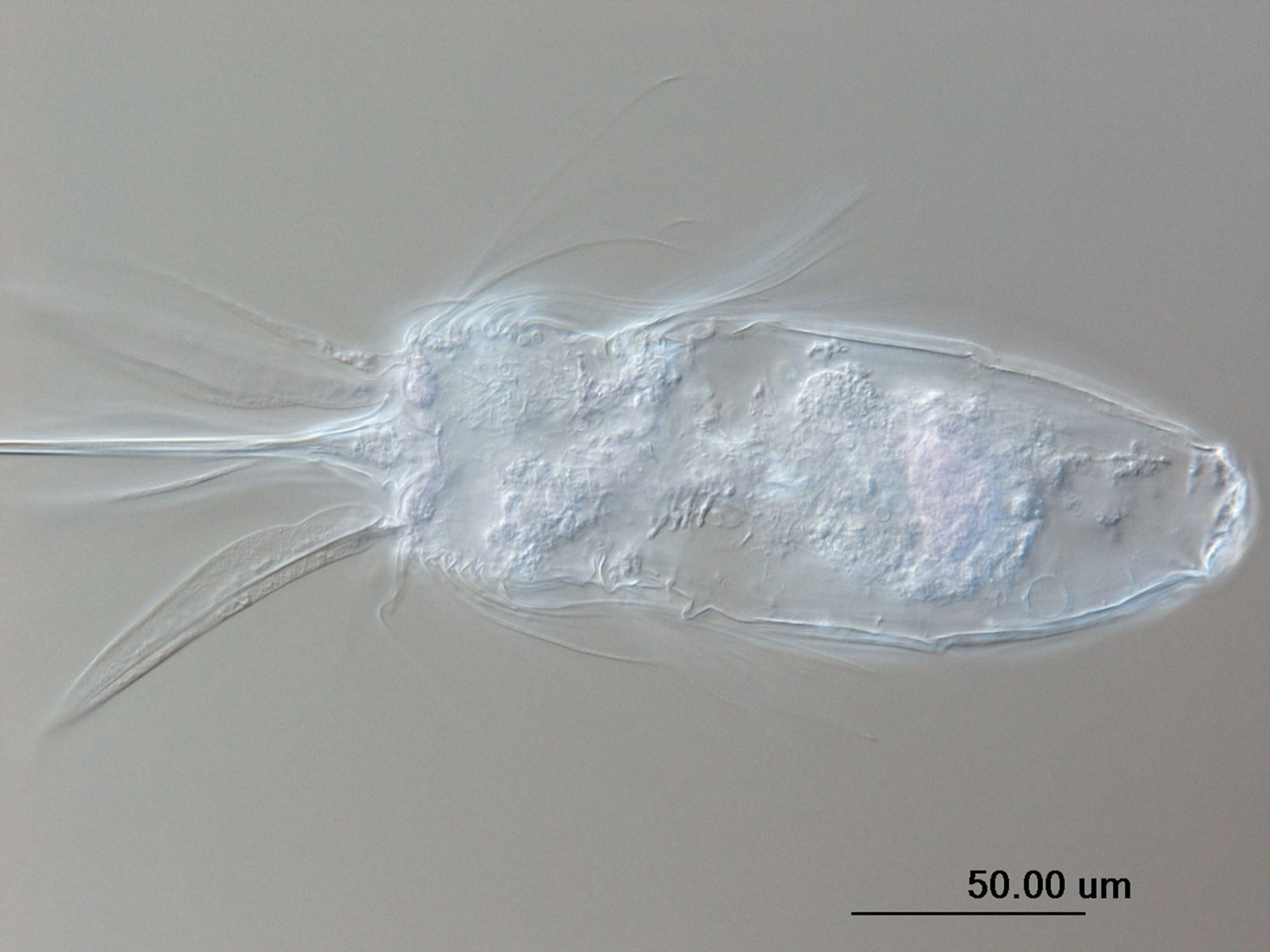

## Slide 4
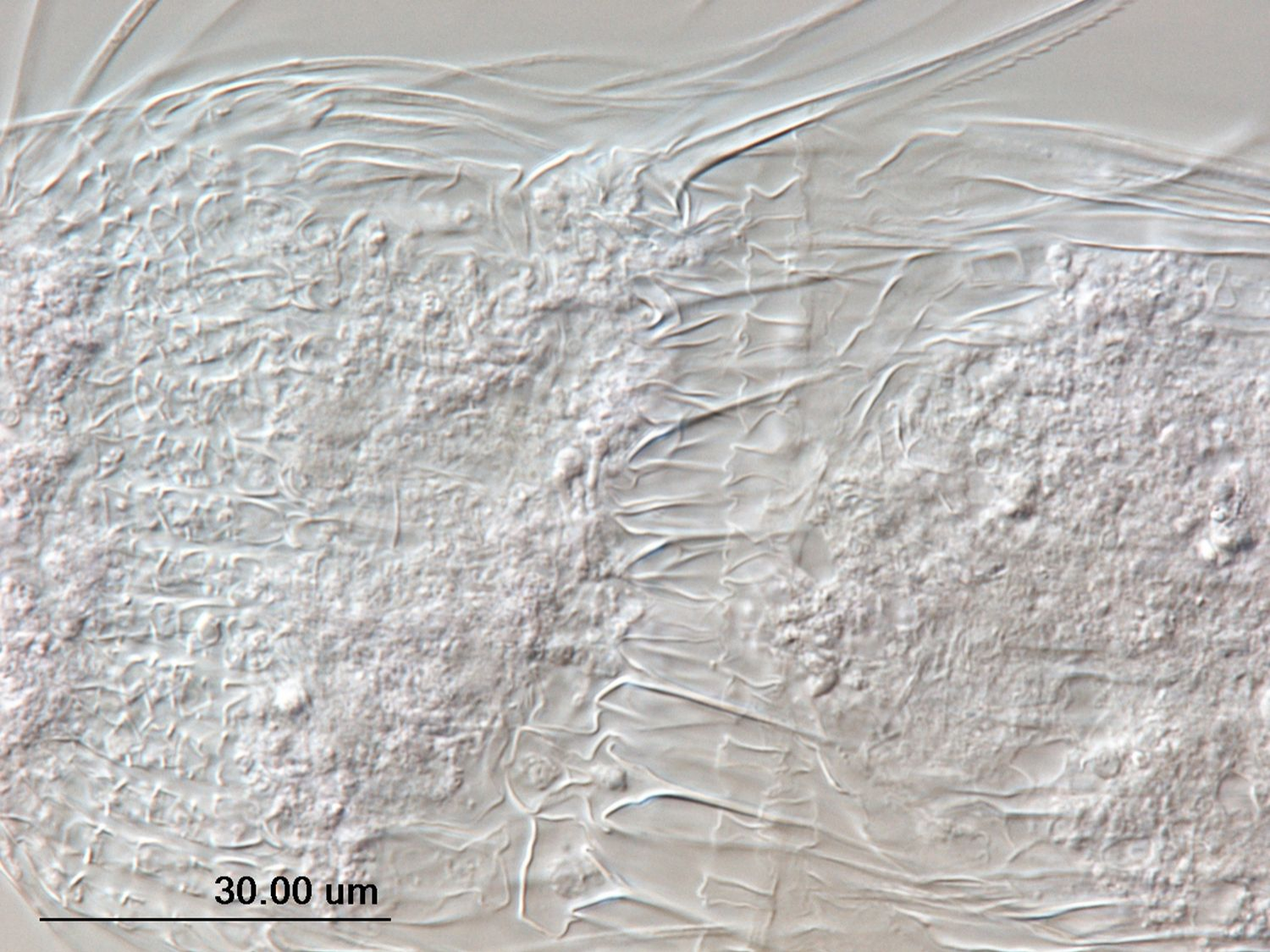

Supplement: Additional file 4: Figure S4. — From the supplemental images of loriciferans, which can be found at the Woods Hole Open Access Server (WHOAS) at http://dx.doi.org/10.1575/1912/7550. Differential interference contrast (DIC) images of a Pliciloricus sp. loriciferan from L’Atalante deep-sea hypersaline anoxic basin, lower halocline (Core sample ID 611 c17). This specimen is shown in Fig. 5c, d in the paper. (PPTX 12024 kb) [file 12915_2015_213_MOESM4_ESM.pptx]
