# Supplementary material for: Metazoans of redoxcline sediments in Mediterranean deep-sea hypersaline anoxic basins
Source: BMC Biol. 2015 Dec 10;13:105. doi: 10.1186/s12915-015-0213-6 (PMC4676161; doi:10.1186/s12915-015-0213-6)
Supplement: Additional file 8: Table S2. — From the supplemental images of loriciferans, which can be found at the Woods Hole Open Access Server (WHOAS) at http://dx.doi.org/10.1575/1912/7550. Nematode densities for each aliquot presented by core designation, per deep-sea hypersaline anoxic basin and habitat. (DOC 14 kb) [file 12915_2015_213_MOESM8_ESM.doc]

Additional file 8: Table S2. Nematode densities for each aliquot presented by core designation, per DHAB and habitat.

| DHAB | Habitat | sample | # aliquots | Aliquot 1 (#/10cm^2^) | Aliquot 2 (#/10cm^2^) | Aliquot 3 (#/10cm^2^) | Aliquot 4 (#/10cm^2^) |
| --- | --- | --- | --- | --- | --- | --- | --- |
| Urania |  |  |  |  |  |  |  |
|  | normoxic, normal saline control | 608-3 | 2 | 0.0 | 62.5 |  |  |
|  | normoxic, normal saline control | 607-4 | 2 | 0.0 | 0.0 |  |  |
|  | normoxic control/halocline transition | 607-8 | 2 | 2303.0 | 5455.0 |  |  |
|  | normoxic control/halocline transition | 607-8C | 2 | 503.9 | 685.0 |  |  |
|  | upper halocline | 607-10 | 4 | 0.0 | 0.0 | 0.0 | 44.3 |
|  | mid-halocline | 608-11A | 4 | 62.5 | 0.0 | 0.0 | 44.3 |
|  | lower halocline | 607-8F | 1 | 0.0 |  |  |  |
|  | lower halocline | 607-8E | 1 | 7.0 |  |  |  |
|  |  |  |  |  |  |  |  |
| Discovery |  |  |  |  |  |  |  |
|  | normoxic, normal saline control | 609-6 | 3 | 2000.0 | 1688.0 | 2125.0 |  |
|  | normoxic, normal saline control | 610-11 | 4 | 0.0 | 62.5 | 0.0 | 4.0 |
|  | upper halocline | 609-7 | 3 | 0.0 | 125.0 | 0.0 |  |
|  | upper halocline | 610-14 | 3 | 928.6 | 2000.0 | 428.6 |  |
|  | mid-halocline | 610-16 | 1 | 7.3 |  |  |  |
|  | mid-halocline | 609-10 H | 1 | 0.0 |  |  |  |
|  | mid-halocline | 610-13 | 1 | 17.3 |  |  |  |
|  | mid-halocline | 610-3 | 2 | 0.0 | 0.0 |  |  |
|  | mid- to lower halocline | 609-1 | 2 | 0.0 | 0.0 |  |  |
|  | lower halocline | 609-14 | 4 | 0.0 | 0.0 | 0.0 | 5.8 |
|  | lower halocline | 610-9 | 3 | 0.0 | 0.0 | 0.0 |  |
|  | lower halocline | 609-4 | entire | 0.0 |  |  |  |
|  | lower halocline | 610-4 | 1 | 1.2 |  |  |  |
|  |  |  |  |  |  |  |  |
| L'Atalante | |  |  |  |  |  |  |
|  | normoxic, normal saline control | 611-3 | 3 | 181.8 | 0.0 | 47.8 |  |
|  | normoxic, normal saline control | 611-8 | 2 | 62.5 | 62.5 |  |  |
|  | upper halocline | 611-5 | 3 | 181.8 | 121.2 | 67.6 |  |
|  | upper halocline | 611-C | 1 | 246.6 |  |  |  |
|  | upper halocline | 611-4 | 1 | 181.8 |  |  |  |
|  | mid-halocline | 611-10 | 4 | 0.0 | 0.0 | 0.0 | 21.9 |
|  | mid-halocline | 611-2 | entire | 0.0 |  |  |  |
|  | lower halocline | 611-14 | 3 | 0.0 | 0.0 | 0.0 |  |
|  | lower halocline | 611-17 | 4* | 0.0 | 0.0 | 0.0 | 2.0 |
|  | lower halocline | 611-18 | entire | 0.0 |  |  |  |

Entire = whole sample analyzed; * equal to entire sample.
